# Validation of the anti-infective potential of a polyherbal *‘Panchvalkal’* preparation, and elucidation of the molecular basis underlining its efficacy against *Pseudomonas aeruginosa*

**DOI:** 10.1186/s12906-019-2428-5

**Published:** 2019-01-17

**Authors:** Chinmayi Joshi, Pooja Patel, Hanmanthrao Palep, Vijay Kothari

**Affiliations:** 1 Institute of Science, Nirma University, Ahmedabad, 382481 India; 2 Dr. Palep’s Medical Research Foundation, Mumbai, India

**Keywords:** Quorum sensing, *Panchvalkal*, Polyherbal, *Pseudomonas aeruginosa*, Transcriptome, *Ayurved*, Antimicrobial resistance, Anti-infective

## Abstract

**Background:**

A *Panchvalkal* formulation (Pentaphyte P-5®) mentioned in ancient texts of Indian traditional medicine was investigated for its anti-infective potential against *Pseudomonas aeruginosa*.

**Methods:**

Effect of the test formulation on bacterial growth and pigment production was evaluated by broth dilution assay. In vivo efficacy was evaluated using *Caenorhabditis elegans* as the model host. Whole transcriptome approach was taken to study the effect of test formulation on bacterial gene expression.

**Results:**

This formulation in vitro was found to be capable of affecting quorum sensing (QS)-regulated traits (pyocyanin, pyoverdine, biofilm) of *Pseudomonas aeruginosa*. In combination with antibiotics, it enhanced susceptibility of the test bacterium to antibiotics like cephalexin and tetracycline. Effect of *Panchvalkal* formulation (PF) on QS-regulated traits of *P. aeruginosa* was not reversed even after repeated exposure of the bacterium to PF. In vivo efficacy of PF was demonstrated employing *Caenorhabditis elegans* as the model host, wherein PF-treated bacteria were able to kill lesser worms than their extract-unexposed counterparts. Whole transcriptome study revealed that approximately 14% of the *P. aeruginosa* genome was expressed differently under the influence of PF.

**Conclusions:**

Major mechanisms through which *Panchvalkal* seems to exert its anti-virulence effect are generation of nitrosative and oxidative stress, and disturbing iron and molybdenum homeostasis, besides interfering with QS machinery. This study is a good demonstration of the therapeutic utility of the ‘polyherbalism’ concept, so common in *ayurved*. It also demonstrates utility of the modern ‘omics’ tools for validating the traditional medicine i.e. ayuromics.

**Electronic supplementary material:**

The online version of this article (10.1186/s12906-019-2428-5) contains supplementary material, which is available to authorized users.

## Background

*Pseudomonas aeruginosa*, an opportunistic human pathogen, has received consideration throughout the history of modern medicine because it remains consistently present among the top pathogens in lists of common hospital ‘superbugs’ around the globe. This organism causes acute pulmonary as well as non-pulmonary infections, chronic lung infections, urinary tract infections, and severe infections in patients with burns and immunosuppression [1–3]. Recently the World Health Organization (WHO) listed carbapenem resistant *P. aeruginosa* as a ‘critical pathogen’ in global priority list of antibiotic-resistant bacteria for which new antibiotics are immediately required [4]. *P. aeruginosa* infection is becoming difficult to treat due to its inherent and acquired resistance to conventional antibiotics and many other antimicrobials. Further, this problem becomes more complex to handle because of the ability of the bacterium to form biofilm and to produce certain virulence factors to cope up with the various stresses employed by the antimicrobials [5]. In *P. aeruginosa*, biofilm formation and virulence factor production are regulated by bacterial cell-to-cell communication mechanism, which is known as Quorum Sensing (QS). QS system of *P. aeruginosa* is complex and it comprises four interconnected signaling systems i.e. *las, rhl, pqs*, and *iqs. las* system is at top of the signaling hierarchy, and is responsible for the production of virulence factors such as protease, elastase, exotoxin, biofilm, etc., Furthermore, environmental factors such as phosphate-depletion, iron starvation, and oxygen deprivation can also modulate the expression of QS-associated genes for survival of pathogen in unfavorable conditions [2, 6].

Since QS controls major virulence mechanisms in bacterial pathogens, interrupting with intercellular communication has revealed a rational strategy to attenuate their virulence without necessarily killing them. Thus, the modulation of QS is being viewed as an attractive alternative to the conventional antimicrobials. Numerous natural compounds and plant extracts have been reported to possess anti-QS activity [6–12], and the need for natural drugs in treatment of various bacterial infections is being felt increasingly, as they offer lesser side effects. Use of natural drugs is the ancient form of healthcare, and *ayurved* is one of the traditional therapeutic systems with a well-documented history of hundreds of years, widely practiced in India. Amongst the drug formulation principles of *ayurved,* ‘Polyherbalism’-combination of medicinal herbs is getting popularity worldwide. It offers some benefits that are not available in modern drugs*.* Polyherbal formulations can serve as potent anti-infective as they contain different phytocompounds that can potentiate the desired biological activity when compatible herbs are formulated together [13].

In present study, we undertook to investigate the effect of a polyherbal preparation described in *ayurved* as ‘*Panchvalkal*’, which has been suggested for treatment of microbial infections viz. vaginal infections and burn wounds [14, 15]. *Panchvalkal* formulation (PF) used in this study contains the barks of different *Ficus* species, mentioned in the treatment of inflammations, abscess and wounds [16]. Various biological activities/ applications of *Panchvalkal* like *vranaprakshalana* (cleaning of wound with a liquid agent), *vranaropana* (healing of ulcer), *shothahara* (anti-inflammatory; reducing oedema or swelling), *upadanshahara* and *visarpahara* (elimination of rash/ blisters typical of sexually transmitted infections) are described in ayurvedic texts namely *Charak Samhita, Bhava Prakasha Nighantu,* and *Sharangadhara Samhita.* In our previous study, we reported QS-modulatory activity of PF against *Chromobacterium violaceum*, *Serratia marcescens* and *Staphylococcus aureus* [17].

We tested this formulation against *P. aeruginosa*, for its QS-modulatory (QSM) potential. Following demonstration of its in vitro QSM potential, we assayed it for in vivo efficacy using the nematode *Caenorhabditis elegans* as a model host. To decipher the molecular basis of its efficacy, whole transcriptome analysis of *P. aeruginosa* exposed to ‘*Panchvalkal*’ was done; its gene expression profile in presence of ‘*Panchvalkal*’ was compared with that in its absence. This study is a demonstration of the role of bacterial QS machinery as an important target for development of new antimicrobials/ anti-infectives, and also of the effective use of modern ‘omics’ for validation of *ayurvedic* prescriptions i.e. ayuromics.

## Methods

### Bacterial strain

Culture of *P. aeruginosa* was obtained from Microbiology Department, M.G. Science Institute, Ahmedabad. Pseudomonas agar (HiMedia, Mumbai) was used for the maintenance of the culture. Antibiotic susceptibility profile of the bacterium was generated using the antibiotic discs- Dodeca Universal-I, Dodeca G-XI Minus, and Icosa Universal-2 (HiMedia, Mumbai). This strain of *P. aeruginosa* was found to be resistant to amoxicillin (30 μg), cefadroxil (30 μg), ampicillin (10 μg), cloxacillin (1 μg), penicillin (10 μg), chloramphenicol (30 μg), cefixime (5 μg), clindamycin (2 μg), and nitrofurantoin (300 μg).

### Test formulation

Capsules of *Panchvalkal* extract (Pentaphyte P5®) containing mixtures of bark extracts of *Ficus bengalensis*, *Ficus religiosa*, *Ficus racemosa*, *Ficus lacor*, and *Albizzia lebbec*, were procured from Dr. Palep’s Medical Research Foundation Pvt. Ltd., Mumbai. Powder contained inside the capsules was dissolved in DMSO (Merck, Mumbai) for bioassay. Starch and talk, which are part of the capsule content (22% *w*/w and 2% w/w respectively), were confirmed to have no effect on measured microbiological parameters.

### Broth dilution assay

Assessment of QS-regulated pigment production by *P. aeruginosa* in presence or absence of the test formulation, was done using broth dilution assay [18]. Organism was challenged with different concentrations (250–1000 μg/mL) of *Panchvalkal* extract. Pseudomonas broth (peptic digest of animal tissue 20 g/L, potassium sulphate 10 g/L, magnesium chloride 1.4 g/L, pH 7.0 ± 0.2) was used as a growth medium. Inoculum standardized to 0.5 McFarland turbidity standard was added at 10%*v*/v, followed by incubation at 37 °C for 22-24 h, with intermittent shaking. Appropriate vehicle control containing DMSO was also included in the experiment, along with abiotic control (containing extract and growth medium, but no inoculum). Catechin (50 μg/mL; Sigma Aldrich) was used as positive control.

### Measurement of bacterial growth and pigment estimation

At the end of the incubation, bacterial growth was quantified photometrically by measuring the culture density at 764 nm wavelength [19]. This was followed by pigment extraction and quantification, as per the method described below for each of the pigment. Purity of each of the extracted pigment was confirmed by running a UV-vis scan (Agilent Cary 60 UV-visible spectrophotometer). Appearance of single major peak (at the λ_max_ reported in literature) was taken as indication of purity.

#### Pyoverdine [20] and pyocyanin [21] extraction

One mL of the culture broth was mixed with chloroform (Merck, Mumbai) in 2:1 proportion followed by centrifugation at 12,000 rpm (15,300 g) (REMI CPR-24 Plus) for 10 min. This resulted in formation of two immiscible layers. OD of the upper water-soluble phase containing yellow-green fluorescent pigment pyoverdine was measured at 405 nm. Pyoverdine Unit was calculated as OD_405_/OD_764_. This parameter was calculated to nullify the effect of change in cell density on pigment production.

The lower chloroform layer containg pyocyanin was mixed with 0.1 N HCl (Merck; at the rate of 20%*v*/v), resulting in a colour change from blue to pink. Absorbance of this pyocyanin in acidic form was measured at 520 nm. Pyocyanin Unit was calculated as OD_520_/OD_764._

### Hemolysis assay [22]

OD of overnight grown culture was standardized to 1.00 at 764 nm. Cell free supernatant was prepared by centrifugation at 15,300 g for 10 min. 10 μL of human blood (sourced from authors’ own body in healthy condition under no antibiotic treatment; collected in heparinized vial) was incubated with this cell free supernatant for 2 h at 37 °C, followed by centrifugation at 800 g for 15 min. 1% Triton X-100 (CDH, New Delhi) was used as positive control. Phosphate buffer saline (PBS; pH 6.8) was used as negative control. OD of the supernatant was read at 540 nm, to quantify the amount of haemoglobin released.

### Assay of bacterial susceptibility to lysis in presence of human serum [23]

Serum was separated by centrifuging blood at 1500 rpm (800 g) for 10 min. Bacterial culture grown in media with and without PF was centrifuged, and the cell pellet was reconstituted in PBS, so that the resulting suspension attains OD_764_ = 1. 200 μl of this bacterial suspension from control or experimental tubes was mixed with 740 μl of PBS and 60 μl of serum. After incubation for 24 h at 37 °C, absorbance was read at 764 nm. DMSO (0.5%*v*/v)-treated cells suspended in PBS served as control, against which OD of the PF-treated cells (serum-exposed) was compared. Tubes containing bacterial cells exposed neither to DMSO nor serum were also included in the experimental set-up, to nullify any interference from autolysis.

### Catalase assay

OD of the overnight grown bacterial culture was adjusted to 1.00 at 764 nm. 400 μL of phosphate buffer was added into a 2 mL vial followed by 400 μL H_2_O_2._ To this 200 μL of the bacterial culture was added, and the mixture was incubated for 10 min. Then 10 μM of sodium azide was added to stop the reaction [24], followed by centrifugation at 12,000 rpm (15,300 g) for 10 min. OD of the supernatant was measured at 240 nm to quantify remaining H_2_O_2_ [25], with phosphate buffer as blank.

### Assay for testing extract’s ability to inhibit biofilm formation, eradicate pre-formed biofilm and affect the biofilm-viability

In this assay, control and experimental, both groups contained nine test tubes. In each group, three subgroups were made. First subgroup of three test tubes in the experimental group contained Pseudomonas broth supplemented with PF (750 μg/mL), whereas remaining six tubes contained Pseudomonas broth with no PF on first day of experiment. All these tubes were inoculated with inoculum (10%*v*/v) standardized to 0.5 McFarland turbidity standard (making total volume in tube 1 mL), followed by incubation at 37 °C for 24 h under static condition, which resulted in formation of biofilm as a ring on walls of the glass tubes. This biofilm was quantified by crystal violet assay [26], preceded by quantification of bacterial cell density and pigment.

Content from the remaining six test tubes from rest of the two subgroups were discarded following cell density and pigment estimation, and then the biofilms remaining on inner surface of these tubes were washed with phosphate buffer saline (PBS; pH 7) to remove loosely attached cells. Now, 2 mL of minimal media (Sucrose 15 g/L, K_2_HPO_4_ 5.0 g/L, NH_4_Cl 2 g/L, NaCl 1 g/L, MgSO_4_ 0.1 g/L, yeast extract 0.1 g/L, pH 7.4 ± 0.2) containing PF (750 μg/mL), was added into each of these tubes, so as to cover the biofilm completely, and tubes incubated for 24 h at 37 °C. At the end of incubation, one subgroup of 3 tubes was subjected to crystal violet assay to know whether any eradication of the pre-formed biofilm has occurred under the influence of PF, and the last subgroup of 3 tubes was subjected to viability assessment through MTT assay.

For the crystal violet assay, the biofilm- containing tubes (after discarding the inside liquid) were washed with PBS in order to remove all non-adherent (planktonic) bacteria, and air-dried for 15 min. Then, each of the washed tubes was stained with 1.5 mL of 0.4% aqueous crystal violet solution for 30 min. Afterwards, each tube was washed twice with 2 mL of sterile distilled water and immediately de-stained with 1500 μL of 95% ethanol. After 45 min of de-staining, 1 mL of de-staining solution was transferred into separate tubes, and read at 580 nm.

For the MTT assay [27], the biofilm- containing tubes (after discarding the inside liquid) were washed with PBS in order to remove all non-adherent (planktonic) bacteria, and air-dried for 15 min. Then 900 μL of minimal media was added into each tube, followed by addition of 100 μL of 0.3% MTT [3-(4,5-Dimethylthiazol-2-yl)-2,5-Diphenyltetrazolium Bromide, HiMedia]. After 2 h incubation at 37 °C, resulting purple formazan derivatives were dissolved in DMSO and measured at 540 nm.

### Determination of the effect of PF on antibiotic susceptibility of the test organism

After in vitro assessment of QSM property of the test formulation, effect of this PF on antibiotic susceptibility of the test pathogen was investigated. This investigation was done in two different ways. In one set of experiments, we challenged the test pathogen with PF and antibiotic simultaneously, wherein susceptibility of test pathogens to sub-MIC concentration of different antibiotics in absence and presence of test formulation was assessed through broth dilution assay. In another set of experiments the bacterial cells pre-treated with PF were subsequently challenged with antibiotic. All the antibiotics were procured from HiMedia, Mumbai.

### In vivo assay [28]

In vivo efficacy of the test formulation at the concentration(s) found effective during in vitro screen was evaluated using the nematode worm *Caenorhabditis elegans* as the model host. This worm was maintained on Nematode Growing Medium (NGM; 3 g/L Nacl, 2.5 g/L peptone, 1 M Cacl_2_, 1 M MgSO_4_, 5 mg/mL cholesterol, 1 M phosphate buffer of pH 6, 17 g/L agar-agar) with *E. coli* OP50 as the feed. Worm population to be used for the in vivo assay was kept on NGM plates not seeded with *E. coli* OP50 for three days.

Test bacterium was incubated with the PF for 24 h. Following incubation, OD of the culture suspension was equalized to that of the DMSO control. 100 μL of this bacterial suspension was mixed with 900 μL of the M9 buffer containing 10 worms (L3-L4 stage). This experiment was performed in 24-well (sterile, non-treated) polystyrene plates (HiMedia), and incubation was carried out at 22 °C. Number of live vs. dead worms was counted everyday till 5 days by putting the plate (with lid) under light microscope (4X). Standard antibiotic and catechin treated bacterial suspension were used as positive control. Straight worms were considered to be dead, and lack of movement in the ‘dead’ looking worms was confirmed by tapping the plates. On last day of the experiment, when plates could be opened, their death was also confirmed by touching them with a straight wire, wherein no movement was taken as confirmation of death.

### Whole transcriptome analysis

#### RNA isolation, library preparation, and sequencing

RNA was extracted from bacterial cell pellet using Hi PURA Bacterial RNA Purification kit (HiMedia, USA) followed by measurement of concentration using QubIT and QC analysis using RNA 6000 Nano Bioanalyzer kit. Whole transcriptome library was prepared from QC qualified samples (RIN > 7) using NEB next ultra RNA library preparation kit (NEB). In brief, 10 μg of Total RNA was taken for ribosomal RNA depletion using Ribominus Bacteria kit module (Invitrogen Inc., USA). The rRNA depleted samples were fragmented using enzymatic method. First strand cDNA synthesis was done using random primers followed by second strand cDNA synthesis, end repair and adapter ligation. The adapter ligated libraries were multiplexed by adding index sequences via amplification. The adapter ligated and indexed libraries were quantitated by QubIT and validated by using Agilent HS kit. Resulting validated libraries were pooled in equimolar ratio and sequenced on NextSeq500 platform (Illumina, USA) using 2x150bp chemistry. The raw data was processed further after necessary quality check with an average Q30 > 70%. All the raw sequence data has been submitted to Sequence Read Archive. Relevant accession no. are SRX2855033 and SRX2855034.

### Genome annotation and functional analysis

#### Read quality check analysis and mapping

QC report of obtained sequences was generated using FastQC application. The high-quality reads were mapped to the reference genome of *P. aeruginosa* PAO1 (NCBI accession no. NC_002516) using RNASeq analysis protocol of CLC Genomics Workbench version 9.0.

#### Differential gene expression analysis

The count data was compared between the samples to identify the differentially expressed genes. The number of reads mapping to each gene was summarised as count data. The count data was first normalised by applying quantile normalization tool in CGWB version 9.0. The count data was further statistically analysed using Kal’s Z-test integrated into CGWB. Genes exhibiting *p* value ≤0.05 were filtered as either up- or down- regulated genes, which were then looked for in panther database for gene ontology classification.

### Statistical analysis

All the experiments were performed in triplicate, and measurements are reported as mean ± standard deviation (SD). Statistical significance of the data was evaluated by applying *t*-test using Microsoft Excel®. *p* values ≤0.05 were considered to be statistically significant.

## Results

### In vitro studies

#### PF modulates the production of QS-regulated pigments

**Fig. 1:**
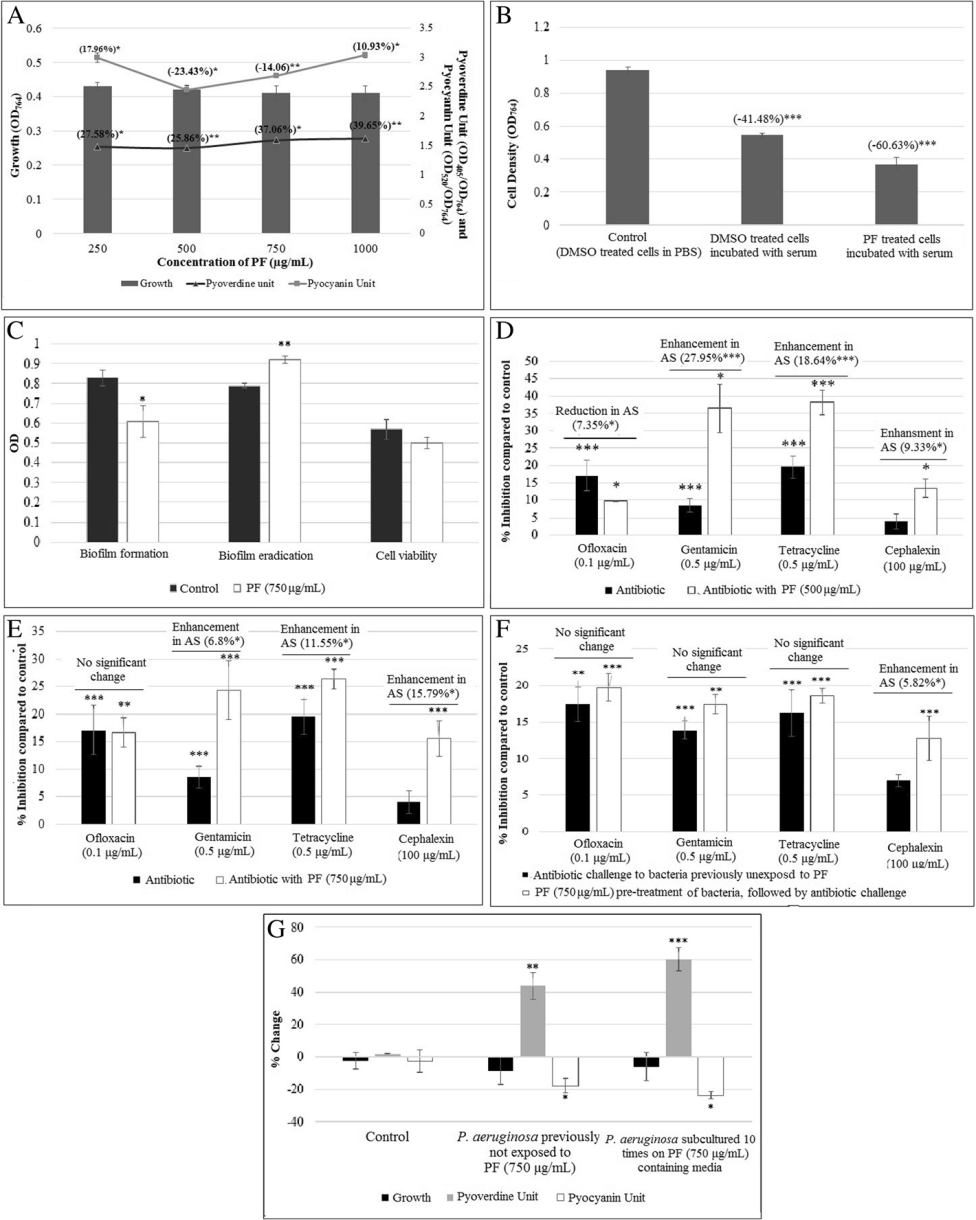
Effect of *Panchvalkal* formulation (PF) on various traits of *P. aeruginosa*, in vitro*.* **a**. Effect of PF on growth and QS-regulated pigment production in *P. aeruginosa.* **b**. PF enhances susceptibility of *P. aeruginosa* to lysis in presence of human serum. **c**. Effect of PF (750 μg/mL) on *P. aeruginosa* biofilm formation, eradication, and viability. **d**. *P. aeruginosa* challenged with PF (500 μg/mL) and antibiotic together. **e**. *P. aeruginosa* challenged with PF (750 μg/mL) and antibiotic together. **f**. *P. aeruginosa* challenged with antibiotic following pre-treatment with PF. **g**. Effect of PF on *P. aeruginosa* growth, pyoverdine unit, and pyocyanin unit remained unaltered after repeated exposure to PF. Bacterial growth was measured as OD_764_; OD of pyoverdine was measured at 405 nm, and Pyoverdine Unit was calculated as the ratio OD_405_/OD_764_ (an indication of pyoverdine production per unit of growth), Pyocyanin Unit was calculated as the ratio OD_520_/OD_764_ (an indication of pyocyanin production per unit of growth); ‘Control’ in this figure is the ‘vehicle control’ representing the % change values in comparison to the ‘growth control’ i.e. tube containing only growth medium plus organism, but no DMSO; 0.5% *v*/v DMSO used as ‘vehicle control’ did not affect biofilm of the bacterium; Crystal violet assay was performed to measure biofilm formation, and biofilm eradication, followed by the measurement of OD at 580 nm; Cell viability in biofilm was estimated through MTT assay, wherein OD was measured at 540 nm; *p < 0.05, **p < 0.01, ***p < 0.001

Pyoverdine production was enhanced at all the test concentrations, whereas pyocyanin production was enhanced at the lowest (250 μg/mL) and highest (1000 μg/mL) test concentrations, and reduced at the intermediate concentrations of PF (Fig. 1a). Effect of PF on QS-regulated pigments in *P. aeruginosa* was not found to be dose-dependent. Such dose-independent response may be for the reason that same plant compounds present in a given extract can interact differently at different concentrations, to produce different biological effect. Bacterial growth was not affected significantly at any of the test concentrations, and hence the effect of PF on *P. aeruginosa* can be said to be purely quorum-modulatory. In fact, an ideal quorum modulator is expected to exert its effect on susceptible pathogens with minimum or no effect on growth [29]. Increased pyoverdine production in the PF-exposed *P. aeruginosa* can be taken as an indication of disturbance of iron homeostasis, as pyoverdine is a known siderophore [30]. Since pyocyanin, whose production was reduced at 500 and 750 μg/mL PF, is an important virulence factor [31], these two concentrations were chosen for further experiments.

### PF affects the catalase activity, and haemolytic potential of the bacterium

QS system of *P. aeruginosa* has been reported to be involved in regulation of antioxidant enzymes like catalase, superoxide dismutase, etc. [32]. Organism can be expected to be under stress in presence of an effective QSM. Hence, we estimated catalase activity of *P. aeruginosa* in absence and presence of PF, and found it to be enhanced by PF to a minor but statistically significant extent (Table 1). Higher catalase activity can be taken as an indication of the organism facing oxidative stress.

**Table 1:** Effect of PF on catalase and haemolytic activity of *P. aeruginosa*

Concentration (μg/mL)	Catalase activity (% change) (Mean ± SD)	Haemolytic activity (% change) (Mean ± SD)
500	1.52** ± 0.67	−8.45* ± 3.48
750	2.14*** ± 0.24	− 15.58* ± 5.20

*p < 0.05, **p < 0.01, ***p < 0.001; ‘-‘sign indicates a decrease over control; DMSO in ‘vehicle control’ tube had no effect on catalase and haemolytic activity of the bacterium

Hemolytic potential, another clinically relevant virulence trait of many infectious bacteria including *P. aeruginosa* [33], was found to be curbed in the range of ~ 8–16% upon exposure of this pathogen to PF (Table 1).

### PF enhances susceptibility of *P. aeruginosa* to lysis in presence of human serum

PF enhanced *P. aeruginosa* killing by ~ 19% when incubated with freshly isolated human serum (Fig. 1b), indicating ability of *Panchvalkal* to impair *P. aeruginosa* resistance to human serum. This can possibly be an additional mechanism by which *Panchvalkal* may confer protection to infection inside human body. This lysis-enhancing trait of PF can be of help to human immune system while fighting infection. We may speculate (however, it needs to be validated experimentally) that the cell surface hydrophobicity of *P. aeruginosa* perhaps gets modulated, when challenged with PF, as the surface hydrophobicity has been shown to be an important determinant for bactericidal action of human serum against *P. aeruginosa* [34]. It may be noted that enhancing the sensitivity of *P. aeruginosa* to serum, through fever induction has been known to be one of the host defense mechanisms against such bacterial infections [35].

### PF inhibits biofilm formation, but does not reduce its viability

PF (750 μg/mL) inhibited biofilm formation in *P. aeruginosa* by 26.50% (Fig. 1c). However, it could not eradicate pre-formed biofilm *P. aeruginosa* biofilm. On the contrary, OD of the crystal violet trapped by biofilm in eradication assay was 16.45% higher in the PF-treated culture, which is difficult to explain. Similar increase in biofilm mass of cinnamaldehyde-treated *Staphylococcus aureus* was reported by Ferro et al., 2016 [23], which they attributed to accumulation of dead cells inside biofilm matrix. However, similar explanation can not be applied to results of this study, as PF had no effect on viability of *P. aeruginosa* biofilm (Fig. 1c). It may be speculated that PF applied on planktonic cells is inhibiting biofilm formation, whereas that applied on biofilm is somehow promoting the biofilm matrix synthesis. In fact, few genes (*pslK, retS*) associated with polysaccharide synthesis/ pilus expression were found to be up-regulated in the whole transcriptome study described in later part of the paper.

### PF modulates the susceptibility of *P. aeruginosa* to different antibiotics

To investigate whether PF can alter antibiotic susceptibility profile of *P. aeruginosa*, we took four antibiotics (ofloxacin, gentamicin, tetracycline, and cephalexin) belonging to different classes i.e. fluoroquinolone, aminoglycoside, tetracyclines, and cephalosporins. PF (500 μg/mL), when used together with antibiotic, made *P. aeruginosa* more susceptible to all the test antibiotics, except ofloxacin. Against latter, its susceptibility was reduced a bit (~ 7%), however this reduction in susceptibility disappeared when PF was used at higher concentration (750 μg/mL) along with antibiotic. Magnitude of alteration in susceptibility to all the tested antibiotics differed at both the PF concentrations used (Fig. 1d-e). These results indicate the potential of *Panchvalkal* as an adjuvant to conventional antibiotic therapy, as already indicated in one of our previous reports [36]. PF pre-treatment of the bacterium before being challenged with these antibiotics did not affect its susceptibility, except in case of cephalexin (Fig. 1f).

### In vivo study

#### PF confers survival benefit on *C. elegans*, when challenged with *P. aeruginosa*

**Fig. 2:**
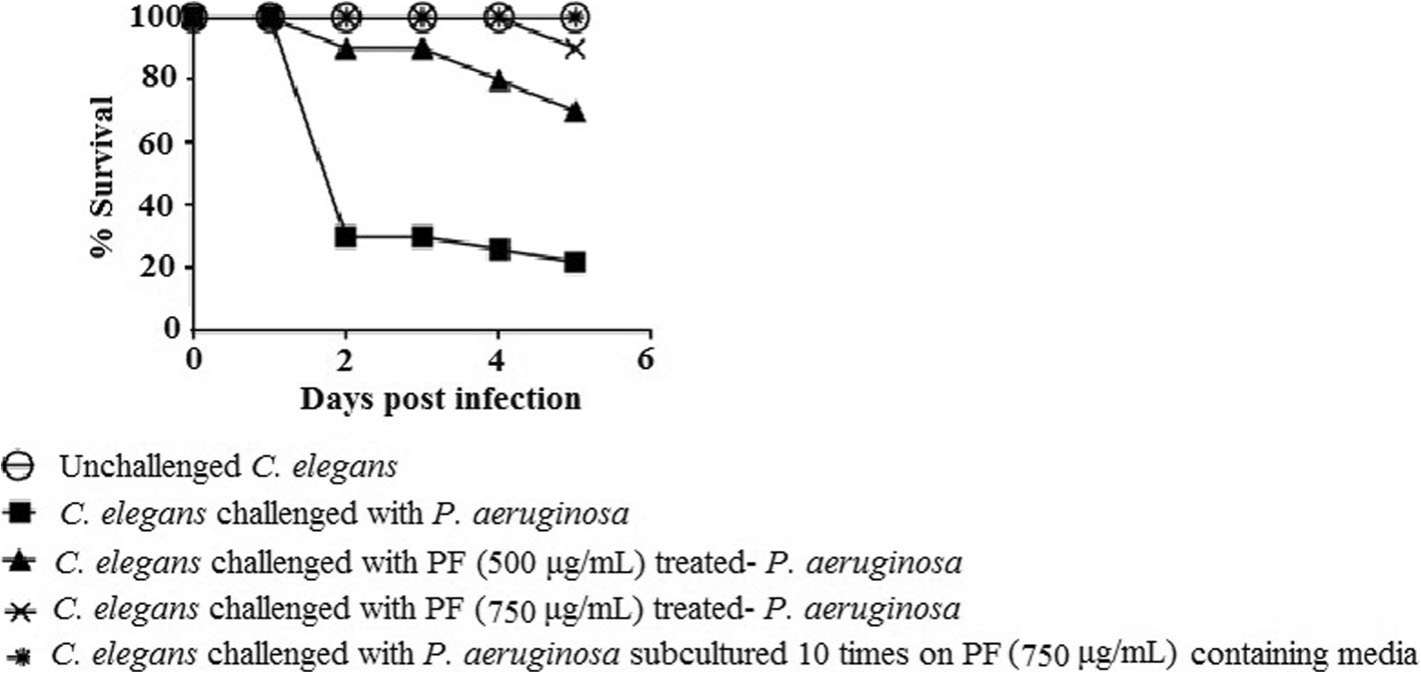
Survival curve showing the protective effect of PF (750 μg/mL) on *C. elegans*, when challenged with *P. aeruginosa.* Catechin (50 μg/mL) and Gentamicin (0.1 μg/mL) employed as positive controls conferred 100 and 80% protection respectively. DMSO present in the ‘vehicle control’ at 0.5%v/v neither affected virulence of the bacterium towards *C. elegans*, nor showed any toxicity towards, worm

After confirming in vitro QSM effect of PF against *P. aeruginosa*, we tested its in vivo efficacy, employing the nematode worm *C. elegans* as the model host. *P. aeruginosa* un-exposed to PF could kill 80% of the worm population by fifth day, wherein 70% were killed just by second day post-infection; whereas that pre-treated with PF (500 or 750 μg/mL) could kill only 30 and 10% worms respectively, by fifth day (Fig. 2). Onset of death in worm population was also delayed by 3 days, in case of *P. aeruginosa* pre-exposed to 750 μg/mL. In fact, the worm population proliferated beyond the start count of 10 (progeny count not recorded).

In addition to challenging *C. elegans* with PF-pre-treated *P. aeruginosa*, we also did an additional experiment, wherein *C. elegans* already infected with *P. aeruginosa* (not previously exposed to PF) were put in liquid medium containing PF (750 μg/mL). Survival benefit conferred by PF on *C. elegans* in this experiment (Additional file 1: Table S5) was statistically identical to that described in preceding paragraph.

Once we witnessed the appreciable anti-infective potential of PF in vitro and in vivo, we proceeded to investigate whether *P. aeruginosa* can develop resistance to this formulation upon repeated exposure. For this, the test pathogen was subcultured 10-times on PF (750 μg/mL)-containing media, and this culture (having multiple exposure to PF) was allowed to infect *C. elegans*. Worm population challenged with this bacterial culture registered a survival of 100% (Fig. 2), equivalent to the ‘control’ worm population not challenged with any pathogenic bacteria. In vitro response of *P. aeruginosa* to PF also was not found to alter, even after repeated exposure to this polyherbal formulation (Fig. 1g). Bacteria may find it difficult to develop resistance against multicomponent preparations, as different components of such formulations can simultaneously affect multiple targets in bacteria, involving more than one mechanisms.

### Whole transcriptome analysis of PF-treated *P. aeruginosa*

A comparative analysis of ‘control’ *P. aeruginosa* culture with that exposed to PF (750 μg/mL; equivalent to 567 μg/mL of plant extract) in context of gene expression at the whole transcriptome level, resulted in identification of 5698 genes; of which 1602 (28.11% of total) were expressed differentially in the PF-treated culture at *p*-value less than or equal to 0.05. However, to have greater confidence in our interpretation of the data, we applied the dual criteria of *p* ≤ 0.05 and fold-change value ≥1.5. In total, there were 787 (13.81% of total) genes satisfying these dual criteria. Of these 787 genes, 389 were down-regulated (Additional file 1: Table S1), and 398 were up-regulated (Additional file 1: Table S2). Fold change values of the up-regulated genes ranged till 16.83, and those for down-regulated ones ranged till 8.77. Additionally a total of 12 genes were found expressed exclusively in the experimental culture (Additional file 1: Table S3). Information on function of all these genes was sourced from KEGG database [37].

## Discussion

**Fig. 3:**
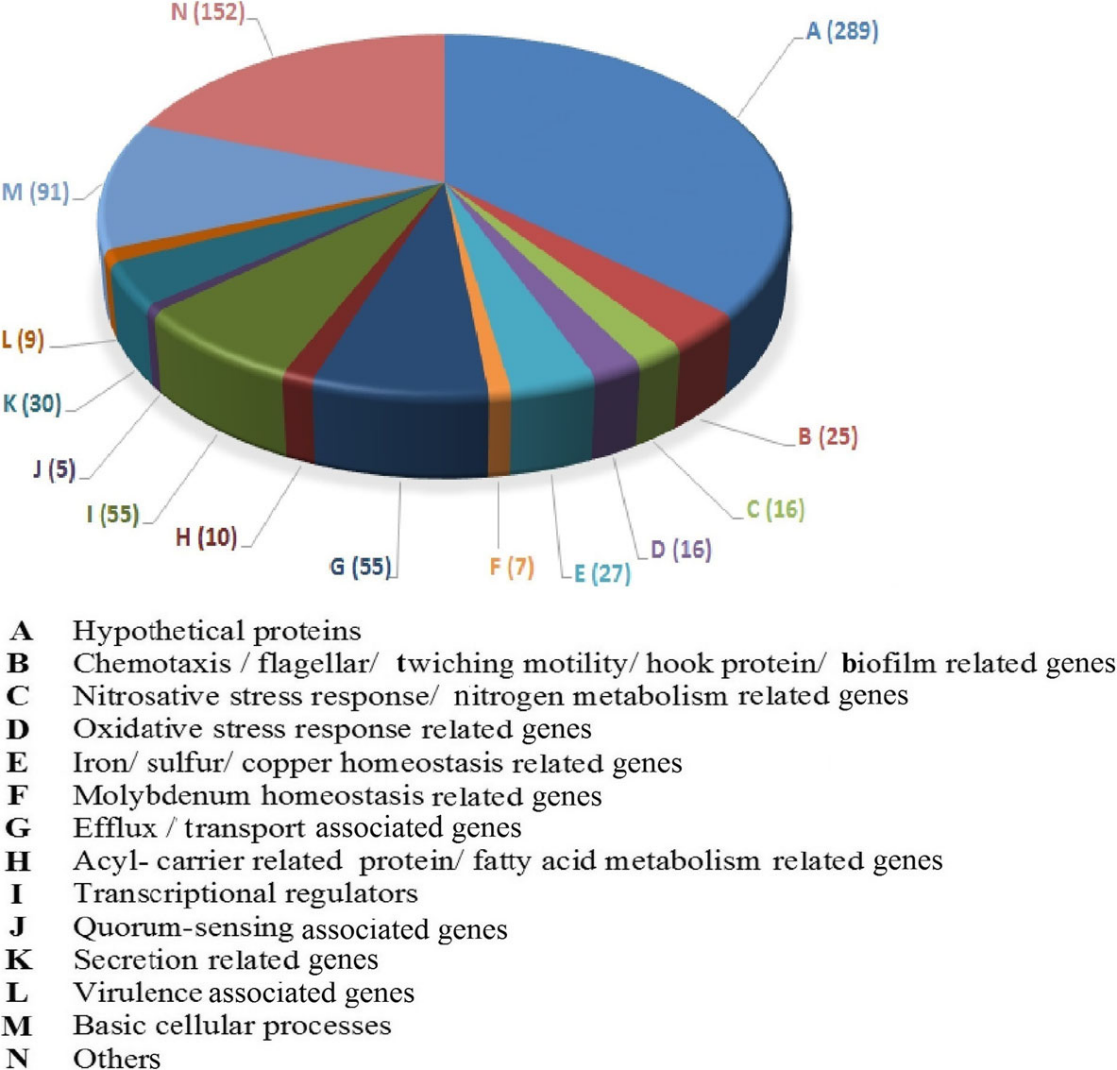
Function-wise categorization of the significantly differentially expressed genes in *Panchvalkal*-treated *P. aeruginosa*

A function-wise categorization of significantly differentially expressed genes is presented in Fig. 3. List of genes in each of these categories is given in Additional file 1: Table S4. Among the top 35 down-regulated genes, five (PA0521, *norB, norC,* PA0525, *nirQ*) were coding for nitric oxide reductase (NOR), whose major function is to detoxify NO generated by nitrite reductase (NIR). Nitric oxide (NO) is a toxic byproduct of anaerobic respiration in *P. aeruginosa*. Though NO toxicity mechanism(s) are not clearly understood, available evidence indicates that NO-derived nitrosative species can damage DNA, and compromise protein function. In denitrifying bacteria, the denitrification pathway is tightly regulated to minimize the adverse effects of NO [38]. *P. aeruginosa* has two nitric oxide (NO)-detoxification enzymes, NOR and flavohemoglobin [39]. The potency of high intracellular NO was demonstrated by showing that a mutant lacking the global QS regulator, *RhlR* (down-regulated in our PF-treated culture by 1.25 fold), committed a metabolic suicide by overproduction of anaerobic NO [38]. Genes involved in denitrification can be expressed even in absence of added nitrate or nitrite [40]. As expression of the enzyme (nitrite reductase) leading to the formation of NO was not shown to be affected in our transcriptome data, we can hypothesize that *P. aeruginosa*’s ability to detoxify NO is compromised under the influence of PF. During our experiments, the test bacterium was incubated under aerobic conditions (without continuous agitation). Denitrification has been observed to occur in presence of oxygen (at dissolved oxygen levels of 1–1.3 mg/L), in many microbial strains including *P. aeruginosa* [41]. Looking at the gene expression profile, it can be said that PF affected nitrogen metabolism in *P. aeruginosa*. Among the genes associated with nitrogen metabolism *napB* and *narH* were respectively up-regulated (5.26 fold) and down-regulated (2.34 fold).

Biofilm formation in many bacteria may be regulated by the signaling molecule NO. Some reports in literature describe an effect of NO on bacterial biofilm formation [42–45]. Endogenously generated NO can have an important role in biofilm regulation. Apparently NO seems to have a broad-spectrum anti-biofilm effect. Our experiments indicated that in presence of PF, *P. aeruginosa* biofilm formation was reduced by 26.50% (Fig. 1c). NO-dependent biofilm regulation in *P. aeruginosa* has been studied extensively. *P. aeruginosa* remain in the biofilm state until exposed to about 0.025–2500 nM NO, at which point they revert to planktonic lifestyle [42]. When exposed to NO concentrations >~ 25 μM, however, biofilm formation was enhanced relative to biofilm formation in the absence of NO. Thus, in *P. aeruginosa*, nanomolar NO can be said to cause biofilm dispersal. One of the top 10 down-regulated genes in our experimental culture, *arsR,* was down-regulated by 6 fold, which acts as a transcriptional regulator for ArsR family, a member of which *PyeR* has been shown to be implicated in regulation of biofilm formation [46].

Response of *P. aeruginosa* to NO is linked to its denitrification abilities. A NO reductase (which was down-regulated in our PF-exposed culture) mutant (Δ*norCB*) exhibits greatly enhanced dispersal [42]. In *P. aeruginosa,* redox signalling regulates the denitrification pathway through a cascade of transcription factors. The global oxygen-sensing regulator ANR (anaerobic regulation of arginine deaminase and nitrate reduction) and DNR (dissimilatory nitrate respiration regulator) regulate a network of genes needed to respond to low oxygen tension and the presence of nitrate, nitrite, and other N-oxides [47–49]. DNR, a member of the FNR family, has been demonstrated to be a heme protein capable of binding NO [50]. In PF-treated culture, DNR was down-regulated by 1.62 fold. Exposure to NO is associated with regulation of intracellular levels of c-di-GMP through the interaction of many diguanylate cyclases (up-regulated by 4.21 fold in PF-treated culture) and phosphodiesterases (down-regulated 1.76 fold in PF-treated culture) [51]. c-di-GMP is a secondary messenger molecule recognized as a key bacterial regulator of multiple processes such as virulence, differentiation, and biofilm formation. Cellular levels of c-di-GMP are modulated by the opposing activities of diguanylate cyclases (DGCs) and phosphodiesterases (PDEs) synthesizing and degrading c-di-GMP, respectively [52]. A chemotaxis transducer *BdlA* has been implicated in c-di-GMP degradation and biofilm dispersal upon NO detection [53, 54]. *BdlA* appears to also respond to many environmental cues in addition to NO. *bdlA* was down-regulated by 1.28 fold under the influence of PF, which might have caused a reduction in the bacteria’s ability to respond to external environmental changes, and also that for chemotaxis. Amongst the chemotaxis associated genes, four (PA4290, PA0175, PA0179, PA0177) were significantly differentially expressed. From the gene expression data, it seems that ability of the PF-treated bacterium to detoxify NO is compromised, whereas such detoxification activity (e.g. NOR) has been reported to be important for virulence expression of this pathogen towards the silkworm *Bombyx mori* [39].

Amongst the significantly down-regulated genes in the PF-treated *P. aeruginosa*, ten were related to secretion systems. Of these, three (PA1702*, pscL, pscT*) were type III secretion system (T3SS) proteins, which are considered as major virulence determinants manipulating eukaryotic host cell responses [55]. Since these proteins are present in a broad range of pathogens, the polyherbal formulation studied by us can be expected to exert a broad-spectrum anti-virulence effect. Earlier we have shown this formulation to be active against multiple gram-positive and gram-negative pathogens viz. *Staphylococcus aureus*, *Chromobacterium violaceum*, and *Serratia marcescens* [17]. Amongst the significantly up-regulated genes, there were 11 secretion-associated proteins, of which one belonged to T3SS, and six were that of Type 6 secretion system (T6SS). Since the secretion-associated genes including those belonging to T3SS/ T6SS were not exclusive to the list of up-regulated or down-regulated genes, we can not have a straight conclusion regarding whether the effect of PF on secretion machinery of *P. aeruginosa* is inhibitory or stimulatory, but it clearly disturbs the secretion machinery on a large scale, as the total number of secretion-associated genes expressed differentially in the PF-treated culture reached 21 (including 2 of T2SS, 4 of T3SS, and 9 of T6SS). T3SS is an important virulence factor of *P. aeruginosa*, shared with many other gram-negative bacteria. It is a hollow molecular needle that transfers effector toxins directly from the bacterium into the host cell cytosol. This complex macromolecular machine works in a heavily regulated manner and is capable of manipulating the host cell in multiple ways [55]. One of the down-regulated T3SS genes *pscL* regulates *pscN*, which is believed to be an ATPase powering the *P. aeruginosa* secretion system [56]. The expression of H1-T6SS genes in *P. aeruginosa* is controlled by the *RetS* sensor [57], which was up-regulated by 1.58 fold. It plays a key role in the reciprocal regulation of virulence factors required for acute versus chronic infection and is postulated to act in concert with two other sensor kinase-response regulator hybrids, *GacS* and *LadS. RetS* also regulates type IV pilus expression and exopolysaccharide synthesis [58]. One of the T6SS secreted protein *VgrG* (PA2373) was down-regulated by 1.63 fold in the PF-exposed culture. VgrG proteins are required for secretion of a genuine H1-T6SS substrate, Tse3, which is one of the seven toxins fired by H1-T6SS. VgrG proteins are not only secreted components but are essential for secretion of other T6SS substrates [57]. Out of the total nine T6SS genes expressed differentially, 6 were up-regulated. The T6SS machinery has been indicated to be expressed upon addition of antimicrobial substances, without the secretion activity necessarily being active. Bacteria may sense presence of antimicrobial substances in their environment, and mount an appropriate self-defense response against them. The T6SS might be one of these defense mechanisms [59]. Hence, the up-regulation of multiple T6SS genes in the PF-exposed culture can be interpreted as one of the defensive responses activated by the bacteria against some of the PF ingredients. However, at the same time, 3 of the down-regulated T6SS genes indicates that in presence of PF, the defensive response of bacteria was not allowed to express fully.

Among the top 20 up-regulated genes, three (*mexC, oprJ,* and *mexD*) were efflux system associated proteins, over-expressed with the fold change values ranging from 4.16 to 16.83. *mexCD-oprJ* is an envelope stress-inducible multidrug efflux operon of *P. aeruginosa*. A gene encoding a homologue of the *NfxB* repressor (up-regulated by 1.69 fold) of this operon, occurs downstream of *oprJ*, and is believed to act as a second repressor of this efflux operon. Expression of PA4596 (up-regulated by 2.50 fold) gets induced under conditions of envelope stress, and thus PF can be said to be capable of putting the susceptible bacterium under envelope stress. MexCD-OprJ is an antibiotic efflux system in *P. aeruginosa,* whose expression is governed by stress, e.g. envelope stress caused by membrane-damaging agents like detergents, organic solvents, hydrocarbons and biocides. This tripartite efflux system accommodates multiple classes of antimicrobials, including clinically relevant antibiotics, and biocides like triclosan and chlorhexidine, and so, its expression promotes multidrug resistance in this organism [60]. However, in our study *P. aeruginosa* failed to develop resistance to PF, even after repeated subculturing on PF-containing medium. Overexpression of MexCD-OprJ was shown to reduce *P. aeruginosa* virulence by promoting its susceptibility to complement-mediated killing [61].

The third top up-regulated (by 10 fold) gene (PA3441/*ssuF*) in PF-treated *P. aeruginosa* was a probable molybdopterin-binding protein. In this culture, *modA* (molybdenum ABC transporter) was found to be down-regulated by 1.59 fold, which is a molybdate transport system permease protein involved in molybldanum homeostasis. In total, 7 genes associated with molybdenum homeostasis were differentially expressed in the PF-treated culture, of which 5 were up-regulated and 2 were down-regulated. Molybdenum homeostasis is required for nitrate utilization, biofilm formation, and virulence expression in *P. aeruginosa* [62]. Nitrate reduction in *P. aeruginosa* is dependent on the availability of oxyanionic form of molybdenum. Down-regulation of *modA* may be a strategy of *P. aeruginosa* to avoid the NO toxicity, as its down-regulation can reduce the cellular molybdate concentrations culminating into an inhibitory effect on nitrate reduction. It can be concluded that under the influence of PF molybdanum homeostasis is disturbed in *P. aeruginosa*, with nitrate reduction being permitted. Conditions that permit nitrate reduction cause inhibition of biofilm formation and alteration in fatty acid composition of *P. aeruginosa*. Disturbance of the molybdenum homeostasis in PF-treated culture can be well corroborated with reduced biofilm formation, altered nitrate reduction, and up-regulation of the genes believed to be involved in responding to the envelope stress, as molybdenum homeostasis has been documented in literature to be important for nitrate reduction, biofilm formation, and cell membrane composition [63].

Among other genes associated with molybdenum homeostasis and/ or cellular processes affected by it, expressed differentially were *narH* (− 2.34 fold), *nirS* (− 2.78 fold), *norC* (− 5.04 fold), *norB* (− 5.44 fold), *napA* (− 1.34 fold), and *napB* (5.26 fold). *narH*, *nirS*, *norCB* are all enzymatic complexes involved in the major dissimilarly nitrate reduction pathway, whereas NapAB codes for a periplasmic nitrate reductase complex. Enzymes of the dissimilatory nitrate reductase pathway require a transition metal cofactor (iron/ copper/ molybdenum) for their activity [63].

Among the differentially expressed genes a good number (33 down-regulated; 22 up-regulated) were those coding for transcriptional regulators. Notable among the down-regulated ones were those (10 in number) part of the family LysR, which regulate transcription of the genes associated with MexEF-OprN efflux system, biofilm formation, virulence, metabolism, QS, and motility. Another gene which can be considered important amongst those affected by PF was *pchB* (down-regulated by 1.91 fold) coding for isochorismate pyruvate lyase. This enzyme is involved in the transformation of isochorismate to pyruvate and salicylate, which is the committed step in biosynthesis of salicylate-based siderophores in many pathogenic bacteria. This is one of the many enzymes involved in synthesis of phenazines and siderophores from chorismate, and being absent from mammals, it can be viewed as a potential target for novel antimicrobials [64].

After having a generalized consideration of the differently expressed genes, we focused particularly on the genes associated with traits assayed by us during in vitro experiments. Pyocyanin is among the most infamous virulence factors of *P. aeruginosa*, whose production was reduced by nearly 14% under the influence of PF. *P. aeruginosa* synthesizes pyocyanin from chorismate through a pathway mediated by phzABCDEFG operon. *phzH* and *phzM*, though just marginally missed passing our cut-off value of 1.5 fold differential expression, their fold change values (− 1.49 and *−* 1.41 respectively) were statistically significant at *p* < 0.05. Both of these are pyocyanin (phenazine) biosynthetic proteins [65, 66]. Pyocyanin is a blue redox-active extracellular phenazine pigment [32], which is produced in response to PQS signalling [67], and acts as a terminal signalling factor in the QS network of *P. aeruginosa* [68]. PQS biosynthesis requires conversion of the central metabolite chorismate to anthranilate by anthranilate synthase. This reaction is also the first step in tryptophan biosynthesis. *P. aeruginosa* possesses two functionally non-redundant anthranilate synthases, TrpEG and PhnAB, of which the latter was down-regulated by 1.32 fold. *kynB* of the kynurenine pathway, which is linked to *P. aeruginosa* virulence as tryptophan is incorporated into the Pseudomonas quinolone signal (PQS) through this pathway, was down-regulated by 3.15 fold. The kynurenine pathway is the main source of anthranilate for PQS production when *P. aeruginosa* is grown in the presence of tryptophan or tryptophan breakdown metabolites [69].

In vitro PF-treated *P. aeruginosa* produced nearly 37% higher pyoverdine. Pyoverdine, a siderophore, is an important virulence factor for *P. aeruginosa* that helps bacteria to survive in iron-limiting conditions [70]. Genes associated with pyoverdine biosynthesis, maturation and transport, *pvdA* (1.28 fold), *pvdF* (1.36 fold), *pvdG* (1.27 fold), *pvdH* (1.24 fold), *pvdP* (1.38 fold), *pvdQ* (1.21 fold), *pvdR* (1.34 fold), were up-regulated significantly, though could not cross the cut-off value of 1.5 fold. *P. aeruginosa* has been reported to produce pyoverdine upon exposure to oxidative stress agent. In general, factors stimulating production of siderophores may be postulated to raise the susceptibility of microorganisms to oxidative damage [71]. Gene expression profile indicates that PF can be believed to be capable of inducing oxidative stress in *P. aeruginosa*, as in total 11 genes involved in oxidative stress response were up-regulated in the PF-treated culture. Of which, two were those coding for catalase i.e. *katA* (1.85 fold) and *katN* (1.67 fold), which is a well-known anti-oxidant enzyme, though the in vitro catalase activity enhancement was smaller (2.14%), albeit statistically significant. Other oxidative stress response genes (PA4172, *ospR*, PA2580, PA0848, *ahpC*, PA3534, PA2826, *nuoK, katA, katN*, PA1266) were upregulated with a fold-change value ranging from 1.62 to 3.93. On the other hand, seven (*nosL, nuoI*, PA3450, PA0942, PA1192, *yrfI*, PA3180) of the oxidative response genes were down-regulated with fold change values ranging from 1.52 to 4.16 which may mean that organism failed to activate its oxidative stress response machinery fully in presence of PF. Oxidative stress stems from generation of the reactive oxygen species (ROS), and potentiation of ROS in disease conditions using small molecules has been considered as drug design strategy for new drug development, since ROS generators may act as effective modulators of virulence [72]. ROS can be generated by destabilization of the iron-sulfur clusters [73], and in our experimental culture, three Fe-S proteins (PA0665, PA1881, PA0185) were differentially expressed passing the dual criteria of *p ≤* 0.05 and fold-change value ≥1.5.

From the hitherto description, we can say that PF makes *P. aeruginosa* face iron starvation and oxidative stress. Here enhanced pyoverdine production seems more likely to be due to oxidative stress, as gene *pchB* coding for isochorismate pyruvate lyase, involved in the conversion of isochorismate into pyruvate and salicylate siderophore, was down-regulated by 1.91 fold, which is likely to lessen the pyochelin mediated iron acquisition in *P. aeruginosa*. Fur, the key regulator of iron metabolism that regulates the expression of iron acquisition and storage systems in response to intracellular iron [74], was down-regulated by 1.16 fold (*p* < 0.00003). It has roles in numerous other aspects of physiology, too. One of the ways, many pathogenic strains employ to fulfil their iron requirement is to source it from the blood cells after hemolysing them. Interestingly, hemolytic potential of this pathogen suffered a ~ 16% downfall owing to PF-treatment. PA3402 coding for a haemolysin secretory protein belonging to the HlyD family was down-regulated by 1.58 fold. Heme contributes to iron-homeostasis in bacteria, which is important for virulence gene expression [75]. Maintenance of iron-homeostasis is an integral part of the metabolism because imbalance in iron metabolism results in ROS generation, which is harmful for the organism [76]. In our experimental culture, genes required for heme biosynthesis *nirS* and heme lyase *ccmH* were down-regulated respectively by 2.78, and 1.94 fold. *bphO* encoding heme oxygenase was down-regulated by 1.76 fold, PA5328 encoding mono-heme cytochrome c was up-regulated by 8 fold, and PA1478 encoding heme-exporter by 3.57 fold. Genes encoding heme acquisition, *hasD* and *hasR* were up-regulated by 2.45 and 1.62 fold respectively. PA4357 coding for ferrous iron transport protein C (2.06 fold), PA0665 encoding iron-sulfur cluster insertion protein (2.05 fold), *exbD1* encoding biopolymer transport protein TolR (2.04 fold), *fepB* for iron-enterobactin transferase (1.76 fold), and PA0192 for iron complex outer membrane receptor protein (1.66 fold) were all down-regulated.

In vitro biofilm formation was inhibited by nearly 25%, when *P. aeruginosa* was challenged with PF (750 μg/mL). Many of the biofilm/polysaccharide synthesis associated genes were differentially expressed in the PF-treated culture. Notable among them are *hutU* (2.46 fold)*,* and *pslK* (2.44 fold). Former is a urocanate hydratase catalyzing histidine degradation, mutation in which is reported to result in reduced biofilm formation [77]. Latter is a polysaccharide biosynthesis protein. Up-regulation of these genes may be a part of *P. aeruginosa*’s response to counteract reduced biofilm formation forced by PF. PA1021 coding for enoyl-CoA hydratase, required for synthesis of the biofilm dispersion autoinducer cis-2-decenoic acid was significantly up-regulated (2.88 fold). Among the relevant down-regulated genes were: flagellar assembly proteins *flgE* (1.55 fold) and *fliE* (1.53 fold)*,* twitching motility protein *pilU* (1.52 fold), pili biogenesis protein *pilC* (1.59 fold)*,* type IV pili assembly protein *pilE* (2.62 fold), lipopolysaccharide biosynthesis proteins PA4819 coding for polyisoprenyl-phosphate glycosyltransferase (2.30 fold) and PA3430 coding for aldolase (1.61 fold), lipopolysaccharide export system protein *LptA* (1.5 fold), alginate biosynthesis proteins *algE* (3.0 fold) and *alg8* (1.52 fold). In context of the down-regulated flagellar/ twitching motility proteins, it should be noted that flagellar and twitching motility have been shown to be necessary for *P. aeruginosa* biofilm development [78].

This study has employed the nematode worm *C. elegans* as the model host for demonstrating the in vivo anti-infective potential of PF. Cezairliyan et al. [79] identified three phenazine class molecules (1-hydroxyphenazine, phenazine-1-carboxylic acid, and pyocyanin) capable of killing nematodes. In our PF-exposed culture, 3 genes *phzC1* (1.97 fold), *pchB* (− 1.91 fold), PA5357 (1.89 fold) associated with phenazine synthesis were differentially expressed with fold-change values ≥1.5. From among the *P. aeruginosa* PA14 virulence-attenuated genes identified in the *C. elegans* infection model by Feinbaum et al. [80], 7 [PA0745 (1.75 fold), PA0215 *liuA* (1.84 fold), *lasI* (1.57 fold), PA1766 (1.58), PA1665 (*fha2*) (1.95 fold), *phzC1* (1.97), *phzH* (− 1.49 fold)] were differently expressed in our PF-treated culture.

Here, we have shown different traits of *P. aeruginosa* such as pyocyanin and pyoverdine production, hemolytic ability, and biofilm formation getting affected by the test formulation. All these traits, and upto a notable extent virulence too, are controlled by QS [33, 81]. A brief description of the differently expressed QS-associated genes follows. Five of the genes (PA1827, PA0182, *fabI*, PA1470, PA5071) involved in the production of the autoinducer 3-oxo-C_12_-HSL/ C_4_-HSL were down-regulated in the range of 2.02–4.52 fold. These two acylhomoserine lactones are the major components of las and rhl systems respectively [82]. PA1827 (*fabG*), down-regulated by 4.52 fold, has a key role in the bacterial fatty acid synthesis II system in pathogenic microorganisms, which has been viewed as an attractive drug target, since fatty acid synthesis is a primary metabolic pathway, and a wide range of cellular processes are dependent on fatty acids [83]. In PF-treated culture, as total of 16 (of which 12 down-regulated) genes associated with acyl-carrier proteins and/or fatty acid metabolism were differentially expressed. QS negative regulator *RsaL*, which has been reported to bind to the *lasI* promoter was down-regulated in our study by 1.50 fold, and this can explain the up-regulation of the *lasI* to almost identical extent (by 1.57 fold). *P. aeruginosa* QS system is connected with other cellular global regulatory networks in a complicated manner, as it is regulated by multiple regulatory factors. Of these many regulatory factors, one is the above mentioned *RsaL*. PA4790 coding for S-adenosylmethionine-dependent methyltransferase involved in production of AI-2, was up-regulated by 2.25 fold. Meijler [84] refers QS as the ‘collective nose’ of bacteria to “smell out” environmental conditions and communicate among themselves, which helps them optimize their virulence response. Differences in environmental conditions such as phosphate and iron availability can significantly influence the activation of the different QS systems [85–87], and that seems to be the case with our PF-exposed culture. Nutrients like iron and phosphate can directly influence the activities of the various QS systems and resultant outcomes on the secretion of virulence factors such as pyocyanin. Quite a few genes in the PF-challenged *P. aeruginosa*, associated with phosphate acquisition/ transport/metabolism viz. PA3383 (6.33 fold ↑), PA2548 (1.55 fold ↑), PA3377 (3.45 fold ↓), and PA0450 (1.60 fold ↓) were differentially expressed.

We tried to see whether any notable overlap can be detected among the genes expressed differentially upon PF-treatment, with those reported in *P. aeruginosa* challenged with other natural products. Such a comparison with the transcriptome of 6-gingerol-treated- *P. aeruginosa* revealed that 96 of the genes reported by them [6] to be differentially expressed, matches with our list of such genes. Little overlap was there with reports describing effect of furanone C-30 [88] or curcumin [32] on *P. aeruginosa* genes/ proteins; number of matching entities being only 4 (PA1665, PA1662, PA1668, *chiC*) and 9 respectively. This however is not surprising, as polyherbal formulations like one used in our study are likely to have more intracellular targets, as against any single molecule.

## Conclusion

**Fig. 4:**
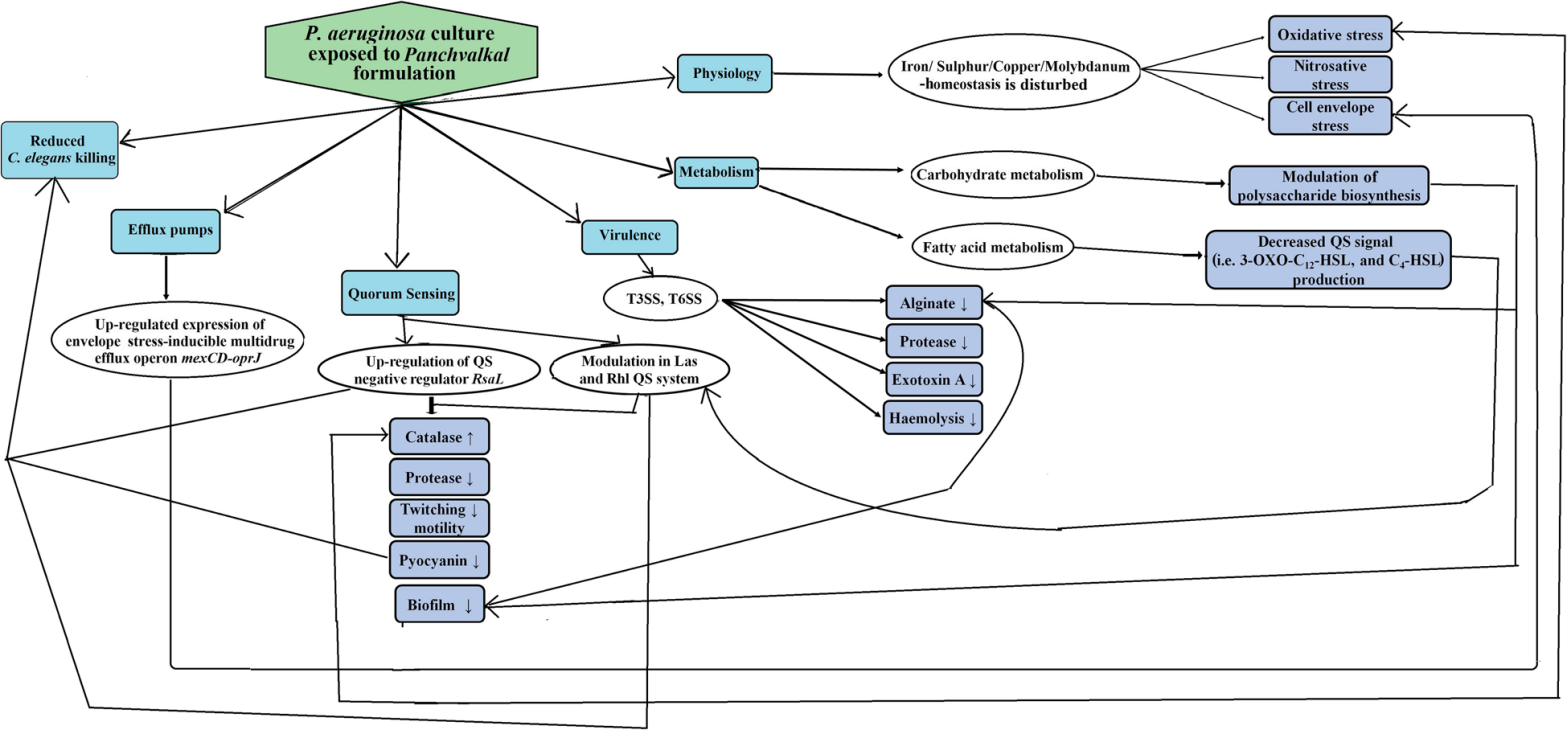
Overall schematic explaining the anti-infective effect of *Panchvalkal* on *P. aeruginosa.* PF acts as an effective QSM against *P. aeruginosa.* It seems to induce oxidative and nitrosative stress in this pathogen, besides disturbing iron- and molybdenum- homeostasis, curbing down its virulence towards *C. elegans,* notably. For detailed discussion, please refer to the text

This study has generated scientific evidence regarding anti-infective potential of the polyherbal *Panchvalkal* preparation, validating its therapeutic use prescribed in *ayurved*. Here we could demonstrate efficacy of PF against one of the most notorious pathogen *P. aeruginosa*, in vitro as well as in vivo. Any polyherbal formulation is very much likely to have multiple targets inside the susceptible pathogenic bacteria, and this was observed with *Panchvalkal* too, as it could significantly alter the expression of 787 (~ 14% of the genome) genes in the test pathogen. To the best of our awareness, this is the first report describing effect of *Panchvalkal* formulation on gene expression of any bacterial pathogen at the whole transcriptome level. The major mechanisms through which PF seems to exert its anti-virulence effect on *P. aeruginosa* (Fig. 4) are by generating oxidative and nitrosative stress, by disturbing the molybdenum- and iron- homeostasis, and by interfering with QS. Since PF exerted its anti-virulence effect without any significant effect on bacterial growth, it can be said not to put a strong selection pressure on this bacterium for resistance development. Even multiple subculturings of this bacterium on PF containing media did not induce resistance, as described in our results of present study. This research report is a good demonstration of the therapeutic utility of the ‘polyherbalism’ concept, so common in *ayurved*, and also of how effective the modern ‘omics’ tools are in validating the traditional medicine i.e. ayuromics. Specifically tailored cocktails of virulence modulators are likely to be more effective solutions in vivo than single agent treatments.

## Additional file

Additional file 1: **Table S1.** List of down-regulated genes. **Table S2.** List of up-regulated genes. **Table S3.** Genes expressed exclusively in PF-exposed *P. aeruginosa* culture. **Table S4.** Category-wise list of genes expressed differentially in PF-treated *P. aeruginosa.* **Table S5.** A comparison of efficacy of *Panchvalkal* when it is used on *P. aeruginosa* before infecting *C. elegnas*, versus, when it is used on *C. elegans* already infected by *P. aeruginosa* having no previous exposure to the *Panchvalkal* extract. (DOCX 91 kb)

## Abbreviations

PF: *Panchvalkal* formulation
QS: Quorum Sensing
QSM: Quorum sensing modulatory
