# Supplementary material for: Validation of the anti-infective potential of a polyherbal ‘Panchvalkal’ preparation, and elucidation of the molecular basis underlining its efficacy against Pseudomonas aeruginosa
Source: BMC Complement Altern Med. 2019 Jan 17;19:19. doi: 10.1186/s12906-019-2428-5 (PMC6335721; doi:10.1186/s12906-019-2428-5)
Supplement: Supplementary file 1 — Table S1. List of down-regulated genes. Table S2. List of up-regulated genes. Table S3. Genes expressed exclusively in PF-exposed P. aeruginosa culture. Table S4. Category-wise list of genes expressed differentially in PF-treated P. aeruginosa. Table S5. A comparison of efficacy of Panchvalkal when it is used on P. aeruginosa before infecting C. elegnas, versus, when it is used on C. elegans already infected by P. aeruginosa having no previous exposure to the Panchvalkal extract. (DOCX 91 kb) [file 12906_2019_2428_MOESM1_ESM.docx]

**Validation of the anti-infective potential of a polyherbal *'Panchvalkal'* preparation, and elucidation of the molecular basis underlining its efficacy against *Pseudomonas aeruginosa***

Chinmayi Joshi^1^, Pooja Patel^1,^ Hanmanthrao Palep^2^, Vijay Kothari^1*^

^1^Institute of Science, Nirma University, Ahmedabad- 382481, Gujarat, India

^2^Dr. Palep’s Medical and Research Foundation, Mumbai, India

^*^correspondence: [vijay.kothari@nirmauni.ac.in](mailto:vijay.kothari@nirmauni.ac.in); [vijay23112004@yahoo.co.in](mailto:vijay23112004@yahoo.co.in)

**Additional file**

Genes listed in Table S1-S2 are only those which got down/up-regulated by a fold change ≥1.5, at p≤0.05, and they have been arranged in decreasing order of fold change. Information on functions of these genes was obtained from Kyoto Encyclopedia of Genes and Genomes (KEGG) [http://www.genome.jp/dbget-bin/get_linkdb?-t+genes+gn:T00035].

**Table S1: List of down-regulated genes**

| **Sr. No.** | **Feature ID/ Gene** | **Coding for** | **Fold Change** | ***p*-value** |
| --- | --- | --- | --- | --- |
|  | PA0521 | Nitric oxide reductase NorE protein | -8.76 | 2.34E-10 |
|  | PA4962 | Inner membrane protein | -8.40 | 0.0001 |
|  | PA2182 | Hypothetical protein | -7.75 | 0.001 |
|  | PA2607 | tRNA 2-thiouridine synthesizing protein B | -6.75 | 0.003 |
|  | PA0691 | Transposase | -6.50 | 0.04 |
|  | *arsR* | ArsR family transcriptional regulator | -6.00 | 0.05 |
|  | PA2980 | Hypothetical protein | -5.88 | 7.77E-05 |
|  | *norB* | Nitric oxide reductase subunit B | -5.44 | 0 |
|  | *norC* | Nitric oxide reductase subunit B | -5.04 | 0 |
|  | PA1827 | 3-oxoacyl-[acyl-carrier protein] reductase | -4.52 | 3.77E-06 |
|  | *atuE* | Isohexenylglutaconyl-CoA hydratase | -4.50 | 0.009 |
|  | PA1013.1 | tRNA-Ser | -4.50 | 0.002 |
|  | PA1492 | Hypothetical protein | -4.20 | 0.001 |
|  | PA2085 | Ring-hydroxylating dioxygenase small subunit | -4.16 | 0.01 |
|  | *nosL* | Copper chaperone NosL | -4.15 | 1.82E-05 |
|  | PA0525 | Nitric oxide reductase NorD protein | -4.14 | 0 |
|  | PA1702 | Type III secretion protein Y | -3.66 | 0.03 |
|  | PA1888 | Hypothetical protein | -3.66 | 0.03 |
|  | PA5071 | 16S ribosomal RNA methyltransferase RsmE | -3.57 | 0.001 |
|  | PA2146 | Hypothetical protein | -3.53 | 0.001 |
|  | *nosY* | Cu-processing system permease protein | -3.46 | 0.002 |
|  | PA3377 | Alpha-D-ribose 1-methylphosphonate 5-phosphate C-P lyase | -3.45 | 0.0002 |
|  | PA1211 | Hypothetical protein | -3.40 | 0.01 |
|  | PA0940 | Hypothetical protein | -3.37 | 0.02 |
|  | PA0818 | Hypothetical protein | -3.30 | 0.004 |
|  | PA3033 | Hypothetical protein | -3.25 | 0.0005 |
|  | *kynB* | Arylformamidase (kynurenine formamidase) | -3.15 | 0.0001 |
|  | PA5196 | Hypothetical protein | -3.14 | 0.005 |
|  | PA5181.1 | P34 | -3.11 | 0.001 |
|  | *nuoI* | NADH-quinone oxidoreductase subunit I | -3.08 | 2.73E-08 |
|  | PA4702 | Hypothetical protein | -3.04 | 1.11E-09 |
|  | *algE* | Alginate production protein | -3.00 | 0.003 |
|  | PA0526 | Hypothetical protein | -2.95 | 0 |
|  | PA2180 | Hypothetical protein | -2.94 | 0.004 |
|  | *nirQ* | Nitric oxide reductase NorQ protein | -2.91 | 0 |
|  | PA0252 | Hypothetical protein | -2.90 | 0.03 |
|  | PA2198 | Hypothetical protein | -2.81 | 0.02 |
|  | PA3493 | Electron transport complex protein RnfG \| | -2.80 | 3.64E-05 |
|  | *nirS* | Heme d1 biosynthesis protein | -2.78 | 0 |
|  | PA1866a | Hypothetical protein | -2.77 | 0.05 |
|  | *infA* | Translation initiation factor IF-1 | -2.73 | 4.9E-06 |
|  | PA1879 | Hypothetical protein | -2.71 | 1.94E-05 |
|  | PA0270 | Hypothetical protein | -2.70 | 0.002 |
|  | PA4466 | Phosphoryl carrier protein | -2.68 | 9.2E-06 |
|  | PA0806 | Hypothetical protein | -2.68 | 0.006 |
|  | PA2506 | Hypothetical protein | -2.66 | 0.01 |
|  | *rluA* | Ribosomal large subunit pseudouridine synthase A | -2.66 | 0.001 |
|  | *lldD* | L-lactate dehydrogenase | -2.65 | 4.74E-05 |
|  | PA3323 | Hypothetical protein | -2.63 | 0.04 |
|  | PA3448 | ABC transporter permease | -2.63 | 0.04 |
|  | PA2570.1 | tRNA-Leu | -2.63 | 0.008 |
|  | PA2433 | Hypothetical protein | -2.62 | 1.35E-09 |
|  | *pcaC* | 4-carboxymuconolactone decarboxylase | -2.62 | 0.001 |
|  | *pilE* | Type IV pilus assembly protein | -2.62 | 0.001 |
|  | PA2754a | Hypothetical protein | -2.59 | 0.005 |
|  | PA4881 | Hypothetical protein | -2.57 | 0.02 |
|  | *pscL* | Type III secretion protein L | -2.55 | 0.01 |
|  | PA0682 | HxcX atypical pseudopilin  (general secretion pathway protein K) | -2.55 | 0.001 |
|  | PA2602 | Hypothetical protein | -2.54 | 0.006 |
|  | *pcaH* | Protocatechuate 3,4-dioxygenase | -2.54 | 0.006 |
|  | PA3580 | Cys-tRNA(Pro)/Cys-tRNA(Cys) deacylase | -2.48 | 5.39E-09 |
|  | PA5535 | Hypothetical protein | -2.44 | 3.13E-08 |
|  | PA0179 | Two-component system, chemotaxis family, response regulator CheY | -2.43 | 5.44E-06 |
|  | PA1312 | Transcriptional regulator | -2.42 | 0.0005 |
|  | PA0431 | Hypothetical protein | -2.42 | 0.005 |
|  | PA2039 | Hypothetical protein | -2.42 | 0.01 |
|  | PA5115 | Hypothetical protein | -2.40 | 0.005 |
|  | *gntR* | Transcriptional regulator GntR | -2.37 | 4.44E-16 |
|  | PA2162 | (1->4)-alpha-D-glucan 1-alpha-D-glucosylmutase | -2.37 | 1.51E-05 |
|  | PA3275 | Small multidrug resistance family-3 protein | -2.36 | 0.001 |
|  | PA3274 | Hypothetical protein | -2.36 | 9.66E-05 |
|  | PA2150 | DNA end-binding protein Ku | -2.35 | 0.0006 |
|  | PA1924 | Hypothetical protein | -2.35 | 0.03 |
|  | PA2935 | Hypothetical protein | -2.35 | 0.03 |
|  | *narH* | Respiratory nitrate reductase beta chain | -2.34 | 2.12E-05 |
|  | PA2136 | Hypothetical protein | -2.33 | 0.0003 |
|  | *nadE* | NH3-dependent NAD synthetase | -2.32 | 0.004 |
|  | PA4171 | Protease I | -2.31 | 0.003 |
|  | PA4819 | Polyisoprenyl-phosphate glycosyltransferase | -2.30 | 0.02 |
|  | PA0924 | Hypothetical protein | -2.29 | 1.81E-09 |
|  | PA3880 | Hypothetical protein | -2.28 | 0.0007 |
|  | PA0544 | Hypothetical protein | -2.27 | 2.71E-08 |
|  | PA0087 | Type VI secretion system protein ImpF | -2.26 | 0.03 |
|  | PA0522 | Hypothetical protein | -2.25 | 0.01 |
|  | PA3450 | Antioxidant protein | -2.25 | 0.006 |
|  | PA4360a | Hypothetical protein | -2.22 | 0.04 |
|  | PA0753 | Putative tricarboxylic transport membrane protein | -2.21 | 0.03 |
|  | PA5155 | Polar amino acid transport system permease protein | -2.20 | 0.01 |
|  | PA0952 | Hypothetical protein | -2.20 | 7.23E-05 |
|  | PA0830 | Hypothetical protein | -2.19 | 2.18E-14 |
|  | PA1093 | Flagellar protein FlaG | -2.19 | 0.008551 |
|  | PA2189 | Hypothetical protein | -2.18 | 0.02 |
|  | PA4064 | Putative ABC transport system ATP-binding protein | -2.18 | 0.007 |
|  | *cueR* | Copper efflux regulator | -2.15 | 0.0002 |
|  | PA0515 | Heme d1 biosynthesis protein NirD | -2.14 | 8.23E-05 |
|  |  | 3-oxoacyl-[acyl-carrier protein] reductase | -2.14 | 0.03 |
|  | PA1020 | Acyl-CoA dehydrogenase | -2.14 | 0.007 |
|  | PA0942 | Transcriptional regulator | -2.14 | 0 |
|  | PA1763 | Hypothetical protein | -2.12 | 0.001 |
|  | PA4921 | Hypothetical protein | -2.12 | 0.0001 |
|  | PA0121 | Hypothetical protein | -2.10 | 2E-05 |
|  | *glpD* | Glycerol-3-phosphate dehydrogenase | -2.10 | 1.14E-12 |
|  | PA3859 | Phospholipase/carboxylesterase | -2.09 | 0.0005 |
|  | PA3172 | Phosphoglycolate phosphatase | -2.09 | 6.52E-05 |
|  | PA4106 | Hypothetical protein | -2.09 | 0.03 |
|  | PA0218 | Transcriptional regulator | -2.08 | 0.0003 |
|  | PA3016 | Hypothetical protein | -2.08 | 0.0009 |
|  | *fabI* | enoyl-[acyl-carrier protein] reductase I | -2.08 | 0.03 |
|  | *braZ* | Branched-chain amino acid:cation transporter | -2.08 | 0.0002 |
|  | *moeA1* | Molybdopterin molybdotransferase | -2.08 | 1.48E-06 |
|  | PA4166 | Acetyltransferase | -2.08 | 0.02 |
|  | PA3224 | Hypothetical protein | -2.06 | 6.11E-07 |
|  | PA4357 | Ferrous iron transport protein C | -2.06 | 1.11E-12 |
|  | PA1221 | Hypothetical protein | -2.06 | 0.002 |
|  | PA0665 | Iron-sulfur cluster insertion protein | -2.05 | 9.64E-12 |
|  | PA3459 | Asparagine synthase | -2.05 | 0 |
|  | PA0443 | Nucleobase:cation symporter-1, NCS1 family | -2.05 | 0.01 |
|  | PA0828 | Transcriptional regulator | -2.04 | 0.0009 |
|  | *exbD1* | Biopolymer transport protein TolR | -2.04 | 0.05 |
|  | PA1015 | Transcriptional regulator | -2.04 | 0.004 |
|  | PA3913 | Putative protease | -2.03 | 1.03E-05 |
|  | PA4779 | O-acetylserine/cysteine efflux transporter | -2.03 | 0.02 |
|  | PA1057 | Multicomponent K+:H+ antiporter subunit E | -2.03 | 0.01 |
|  | *ohrR* | Transcriptional regulator | -2.03 | 0.01 |
|  | PA0177 | Purine-binding chemotaxis protein CheW | -2.03 | 0.01 |
|  | PA3847 | Hypothetical protein | -2.03 | 0.01 |
|  | PA0911 | Hypothetical protein | -2.02 | 0.01 |
|  | PA1470 | 3-oxoacyl-[acyl-carrier protein] reductase | -2.02 | 0.003 |
|  | PA3070 | MoxR-like ATPase | -2.01 | 5.1E-07 |
|  | PA5469 | Hypothetical protein | -2 | 0.04 |
|  | *narL* | Two-component system, NarL family, sensor histidine kinase BarA | -1.98 | 2.92E-11 |
|  | *crfX* | Hypothetical protein | -1.98 | 5.96E-05 |
|  | PA1257 | Polar amino acid transport system permease protein | -1.97 | 0.01 |
|  | PA1873 | Zinc transporter | -1.97 | 0.0005 |
|  | PA0160 | Hypothetical protein | -1.96 | 0.02 |
|  | PA1844 | Hypothetical protein | -1.96 | 0.02 |
|  | PA3720 | Hypothetical protein | -1.96 | 0.0003 |
|  | *ribA* | GTP cyclohydrolase II | -1.95 | 2.27E-06 |
|  | PA3786 | Hypothetical protein | -1.95 | 0.008 |
|  | PA2829 | Hypothetical protein | -1.95 | 0.01 |
|  | PA4636a | Hypothetical protein | -1.95 | 0.01 |
|  | PA0119 | Aerobic C4-dicarboxylate transport protein | -1.94 | 1.68E-07 |
|  | PA3403 | Hypothetical protein | -1.94 | 0.0008 |
|  | PA0422 | Hypothetical protein | -1.94 | 0.02 |
|  | *ccmH* | Cytochrome c-type biogenesis protein CcmH | -1.94 | 0.005 |
|  | *nirM* | Cytochrome c | -1.93 | 0.0004 |
|  | PA5055 | Hypothetical protein | -1.93 | 0.003 |
|  | PA5540 | Hypothetical protein | -1.93 | 0.03 |
|  | *dhcB* | 3-oxoacid CoA-transferase subunit B (Dehydrocarnitine CoA transferase) | -1.92 | 1.56E-06 |
|  | PA1507 | Nucleobase: cation symporter-2 | -1.92 | 0.0001 |
|  | PA1398 | Hypothetical protein | -1.91 | 2.47E-05 |
|  | PA0485 | Chloramphenicol-sensitive protein RarD | -1.91 | 9.41E-13 |
|  | PA2544 | Hypothetical protein | -1.91 | 4.56E-05 |
|  | *pchB* | Isochorismate pyruvate lyase | -1.91 | 2.19E-05 |
|  | PA2317 | gamma-glutamylputrescine oxidase | -1.91 | 0.01 |
|  | *cynS* | Cyanate lyase | -1.90 | 0.03 |
|  | PA2206 | Transcriptional regulator | -1.89 | 0.0001 |
|  | PA1206 | Hypothetical protein | -1.89 | 0.02 |
|  | PA3358 | Hypothetical protein | -1.89 | 0.02 |
|  | *hisM* | Histidine transport system permease protease | -1.89 | 0.02 |
|  | PA5179 | Transcriptional regulator | -1.88 | 0.0009 |
|  | *aruH* | Arginine:pyruvate transaminase | -1.88 | 0.003 |
|  | *pcs* | Phosphatidylcholine synthase | -1.88 | 0.002 |
|  | PA4311 | alpha-1,6-mannosyltransferase | -1.88 | 0 |
|  | *cupB3* | Outer membrane usher protein | -1.88 | 6.51E-06 |
|  | PA0322 | Transporter | -1.87 | 0.0002 |
|  | *ccoQ1* | Cytochrome c oxidase cbb3-type subunit IV | -1.87 | 0.01 |
|  | PA0673 | Hypothetical protein | -1.87 | 0.006 |
|  | PA5324 | Transcriptional regulator | -1.87 | 2.09E-05 |
|  | PA2178 | Hypothetical protein | -1.87 | 0.001 |
|  | PA4046 | Hypothetical protein | -1.87 | 0.006 |
|  | PA1184 | LysR family transcriptional regulator, glycine cleavage system transcriptional activator | -1.86 | 0.004 |
|  | *pncB1* | Nicotinate phosphoribosyltransferase | -1.86 | 0.002 |
|  | *dctA* | Aerobic C4-dicarboxylate transport protein | -1.86 | 0.009 |
|  | PA2030 | Hypothetical protein | -1.86 | 0.002 |
|  | *moaC* | Cyclic pyranopterin monophosphate synthase (Molybdenum cofactor) | -1.85 | 0.01 |
|  | PA4451 | Hypothetical protein | -1.85 | 0.0008 |
|  | PA1410 | Putrescine transport system substrate-binding protein | -1.85 | 0.02 |
|  | PA1208 | Hypothetical protein | -1.84 | 0.0003 |
|  | PA3091 | Hypothetical protein | -1.83 | 2.59E-10 |
|  | *cysW* | Sulfate transport system permease protein | -1.83 | 0.004 |
|  | PA1170 | Hypothetical protein | -1.83 | 0.01 |
|  | PA5212 | Hypothetical protein | -1.83 | 4.36E-08 |
|  | PA3965 | Lrp/AsnC family transcriptional regulator, leucine-responsive regulatory protein | -1.82 | 0.01 |
|  | *exsB* | Exoenzyme S synthesis protein | -1.82 | 0.01 |
|  | PA0591 | ApaG protein | -1.82 | 0.01 |
|  | PA4100 | Dehydrogenase | -1.81 | 0.005 |
|  | PA0276 | Hypothetical protein | -1.81 | 0 |
|  | PA2630 | 50S ribosomal protein L16 3-hydroxylase | -1.80 | 4.68E-05 |
|  | PA4913 | Branched-chain amino acid transport system substrate-binding protein | -1.79 | 1.9E-06 |
|  | PA3899 | RNA polymerase sigma-70 factor, ECF subfamily | -1.79 | 0.001 |
|  | *lgt* | Phosphatidylglycerol:prolipoprotein diacylglycerol transferase | -1.79 | 0.0005 |
|  | PA0573 | Hypothetical protein | -1.79 | 0.01 |
|  | *ppgL* | Gluconolactonase | -1.78 | 1.67E-05 |
|  | PA0042 | Hypothetical protein | -1.78 | 0.0009 |
|  | PA2483 | Hypothetical protein | -1.78 | 0.001 |
|  | PA0012 | Hypothetical protein | -1.77 | 0.008 |
|  | *fadH1* | 2,4-dienoyl-CoA reductase (NADPH2) | -1.76 | 1.8E-06 |
|  | PA4108 | Cyclic di-GMP phosphodiesterase | -1.76 | 2.67E-07 |
|  | *bphO* | Heme oxygenas | -1.76 | 0.0003 |
|  | PA1332 | Hypothetical protein | -1.76 | 0.008 |
|  | PA2719 | Hypothetical protein | -1.76 | 0.02 |
|  | *fepB* | Iron complex transport system substrate-binding protein (Iron-enterobactin transferase) | -1.76 | 0.001 |
|  | PA1736 | Acetyl-CoA C-acetyltransferase | -1.75 | 6.19E-10 |
|  | PA0530 | Acetylornithine/N-succinyldiaminopimelate aminotransferase | -1.75 | 2.82E-12 |
|  | PA3912 | Hypothetical protein | -1.75 | 0.001 |
|  | *pelA* | Polysaccharide biosynthesis protein | -1.75 | 0.001 |
|  | *iscS* | Cysteine desulfurase | -1.75 | 2.66E-10 |
|  | *cbpA* | cAMP-binding protein A | -1.75 | 4.05E-05 |
|  | PA3747 | Hypothetical protein | -1.75 | 0.01 |
|  | PA0467 | Hypothetical protein | -1.75 | 0.02 |
|  | PA3413 | Hypothetical protein | -1.74 | 1.65E-10 |
|  | PA4995 | Acyl-CoA dehydrogenase | -1.73 | 0.002 |
|  | PA4471 | Hypothetical protein | -1.73 | 4.15E-05 |
|  | PA2588 | Transcriptional regulator | -1.73 | 0.004 |
|  | PA2296 | NitT/TauT family transport system substrate-binding protein | -1.73 | 0.01 |
|  | PA2758 | Transcriptional regulator | -1.72 | 0.007 |
|  | PA0277 | Putative metalloprotease | -1.72 | 2.58E-12 |
|  | PA3451 | Hypothetical protein | -1.72 | 0.04 |
|  | PA0492 | UPF0271 protein | -1.72 | 0.003 |
|  | PA2919 | Hypothetical protein | -1.71 | 0.001 |
|  | PA3201 | Intracellular septation protein \| | -1.71 | 0.002 |
|  | PA4405 | Hypothetical protein | -1.71 | 0.002 |
|  | *gpuP* | Solute:Na+ symporter, SSS family (3-guanidinopropionate transporter) | -1.71 | 0.01 |
|  | *dhcA* | 3-oxoacid CoA-transferase subunit A | -1.71 | 0 |
|  | PA5030 | MFS transporter, YNFM family, putative membrane transport protein | -1.70 | 0.0001 |
|  | PA0490 | Hypothetical protein | -1.70 | 3.71E-11 |
|  | PA2047 | Transcriptional regulator | -1.70 | 0.01 |
|  | *glcE* | Glycolate oxidase FAD binding subunit | -1.70 | 6.91E-05 |
|  | PA2458 | Hypothetical protein | -1.70 | 0.05 |
|  | PA5275 | CyaY protein (Frataxin-like protein) | -1.69 | 1.76E-11 |
|  | PA2369 | Type VI secretion system protein ImpG | -1.69 | 0.01 |
|  | *aat* | Leucyl/phenylalanyl-tRNA---protein transferase | -1.69 | 0.0002 |
|  | PA2691 | NADH dehydrogenase | -1.69 | 0.005 |
|  | PA3359 | Hypothetical protein | -1.69 | 0.01 |
|  | PA0367 | Transcriptional regulator | -1.69 | 0.001 |
|  | PA5327 | Oxidoreductase | -1.69 | 0.02 |
|  | PA3722 | Hypothetical protein | -1.69 | 0.006 |
|  | PA4045 | Vitamin B12 transport system substrate-binding protein | -1.68 | 4.17E-05 |
|  | PA3881 | Hypothetical protein | -1.68 | 0.04 |
|  | PA3012 | Hypothetical protein | -1.68 | 0.0005 |
|  | PA1131 | MFS transporter, DHA1 family, tetracycline resistance protein | -1.68 | 0.01 |
|  | *lipA* | Triacylglycerol lipase | -1.68 | 0.02 |
|  | PA0537 | LemA protein | -1.68 | 9.87E-05 |
|  | PA2041 | Putrescine importer (Amino acid permease) | -1.68 | 0.001 |
|  | PA3530 | Bacterioferritin-associated ferredoxin | -1.68 | 1.42E-09 |
|  | PA1075 | Hypothetical protein | -1.67 | 0.002 |
|  | PA2284 | Hypothetical protein | -1.67 | 0.005 |
|  | PA3025 | Glycerol-3-phosphate dehydrogenase | -1.67 | 1.84E-06 |
|  | PA1141 | Transcriptional regulator | -1.67 | 0.005 |
|  | PA4382 | Hypothetical protein | -1.67 | 0.0007 |
|  | PA0192 | Iron complex outermembrane recepter protein (TonB-dependent receptor) | -1.66 | 0.003 |
|  | PA4575 | Hypothetical protein | -1.66 | 1.02E-11 |
|  | PA1644 | Hypothetical protein | -1.66 | 0.004 |
|  | PA2207 | Hypothetical protein | -1.66 | 0.03 |
|  | PA3278 | Hypothetical protein | -1.66 | 2.03E-07 |
|  | PA3425 | Hypothetical protein | -1.66 | 0.02 |
|  | PA4185 | Transcriptional regulator | -1.66 | 0.02 |
|  | PA3517 | Adenylosuccinate lyase | -1.66 | 0.01 |
|  | *fis* | Fis family transcriptional regulator, factor for inversion stimulation protein | -1.66 | 0.02 |
|  | *glpR* | DeoR family transcriptional regulator, glycerol-3-phosphate regulon repressor | -1.66 | 0.02 |
|  | *aphA* | Acetylpolyamine aminohydrolase | -1.66 | 0.03 |
|  | PA4536 | Hypothetical protein | -1.65 | 0.004 |
|  | PA1146 | Iron-containing alcohol dehydrogenase | -1.65 | 0.01 |
|  | PA3522 | Gold/copper resistance efflux pump (Resistance-nodulation-cell division (RND) efflux pump) | -1.65 | 3.92E-05 |
|  | PA5284 | Hypothetical protein | -1.65 | 0.009 |
|  | PA2884 | Hypothetical protein | -1.65 | 0.005 |
|  | PA2662 | Uncharacterized protein involved in response to NO | -1.64 | 8.5E-05 |
|  | *pcpS* | Enterobactin synthetase component D | -1.64 | 0.0001 |
|  | PA2336 | Hypothetical protein | -1.64 | 0.05 |
|  | PA5303 | Hypothetical protein | -1.64 | 0.0002 |
|  | PA0736 | Hypothetical protein | -1.64 | 0.01 |
|  | PA2325 | Hypothetical protein | -1.64 | 0.03 |
|  | PA4155 | Hypothetical protein | -1.64 | 2.22E-06 |
|  | PA0859 | Putative protein-disulfide isomerase | -1.64 | 0.001 |
|  | PA2270 | Transcriptional regulator | -1.64 | 0.007 |
|  | PA3307 | Hypothetical protein | -1.64 | 7.23E-09 |
|  | *rhdA* | Thiosulfate/3-mercaptopyruvate sulfurtransferase | -1.63 | 0.03 |
|  | PA2271 | Acetyltransferase | -1.63 | 0.01 |
|  | PA2373 | Type VI secretion system secreted protein VgrG | -1.63 | 0.01 |
|  | PA2930 | Transcriptional regulator | -1.62 | 0.003 |
|  | PA2355 | FMNH2-dependent monooxygenase | -1.62 | 0.02 |
|  | PA1192 | tRNA 2-thiocytidine biosynthesis protein TtcA | -1.62 | 0.01 |
|  | PA1790 | tRNA-(ms[2]io[6]A)-hydroxylase | -1.62 | 0.002 |
|  | PA0481 | Hypothetical protein | -1.62 | 0.02 |
|  | PA2135 | Transporter | -1.62 | 0.03 |
|  | PA4692 | Methionine sulfoxide reductase catalytic subunit | -1.62 | 0.005 |
|  | PA2606 | tRNA 2-thiouridine synthesizing protein C | -1.62 | 0.009 |
|  | *dnr* | Positive regulation of nitrogen compound metabolic process | -1.62 | 3.19E-05 |
|  | PA5518 | Potassium efflux transporter | -1.61 | 0.001 |
|  | PA3430 | Aldolase | -1.61 | 0.006 |
|  | PA3678 | TetR/AcrR family transcriptional regulator, mexJK operon transcriptional repressor | -1.61 | 5.7E-07 |
|  | PA5428 | LysR family transcriptional regulator, hypochlorite-specific transcription factor HypT | -1.61 | 0.003 |
|  | PA5023 | Hypothetical protein | -1.61 | 0.0001 |
|  | PA0038 | Hypothetical protein | -1.61 | 1.04E-12 |
|  | PA0043 | Hypothetical protein | -1.61 | 0.02 |
|  | PA1764 | Hypothetical protein | -1.61 | 0.02 |
|  | PA0529 | Hypothetical protein | -1.61 | 5.96E-06 |
|  | PA0242 | 4-hydroxyphenylpyruvate dioxygenase | -1.61 | 0.006 |
|  | *aph* | Aminoglycoside resistance gene | -1.61 | 0.009 |
|  | PA0457.1 | Hypothetical protein | -1.60 | 0.01 |
|  | PA1711 | Hypothetical protein | -1.60 | 0.02 |
|  | PA0450 | Inorganic phosphate transporter, PiT family (Phosphate transporter) | -1.60 | 0.002 |
|  | PA3521 | Outer membrane protein, multidrug efflux system | -1.60 | 0.01 |
|  | PA4136 | MFS transporter, DHA1 family, multidrug resistance protein | -1.60 | 0.001 |
|  | *metY* | O-acetylhomoserine (thiol)-lyases | -1.60 | 0.002 |
|  | PA0257 | Putative transposase | -1.60 | 0.004 |
|  | PA0325 | ABC transporter permease | -1.60 | 0.04 |
|  | *gpuR* | Transcriptional activator | -1.60 | 0.001 |
|  | PA3306 | Hypothetical protein | -1.60 | 4.01E-05 |
|  | PA1870 | Hypothetical protein | -1.60 | 0.01 |
|  | PA0120 | GntR family transcriptional regulator, transcriptional repressor for pyruvate dehydrogenase c | -1.59 | 3.78E-07 |
|  | PA1264 | Transcriptional regulator | -1.59 | 0.0008 |
|  | PA2801 | Acyl-CoA thioester hydrolase | -1.59 | 0.02 |
|  | PA3404 | Outer membrane protein, protease secretion system | -1.59 | 0.008 |
|  | PA1392 | Hypothetical protein | -1.59 | 0.003 |
|  | *moaE* | Molybdopterin synthase catalytic subunit | -1.59 | 0.02 |
|  | PA1484 | Transcriptional regulator | -1.59 | 0.003 |
|  | *pilC* | Pseudogene | -1.59 | 2.12E-08 |
|  | *modA* | Molybdenum ABC transporter | -1.59 | 0.04 |
|  | *metR* | LysR family transcriptional regulator, regulator for metE and metH | -1.59 | 0.009 |
|  | PA5519 | Acyl-CoA thioesterase | -1.58 | 0.001 |
|  | *gltR* | Two-component response regulator GltR | -1.58 | 0.01 |
|  | PA5219 | Hypothetical protein | -1.58 | 9.85E-05 |
|  | PA3854 | GIY-YIG nuclease superfamily protein | -1.58 | 0.02 |
|  | PA3402 | HlyD family secretion protein | -1.58 | 0.009 |
|  | PA2157 | Hypothetical protein | -1.58 | 0.02 |
|  | PA1037 | Hypothetical protein | -1.58 | 1.73E-06 |
|  | PA1334 | N-ethylmaleimide reductase | -1.58 | 0.02 |
|  | PA0216 | Malonate transporter MadM | -1.57 | 0.01 |
|  | PA0688 | Alkaline phosphatase L | -1.57 | 0.01 |
|  | PA3684 | Hypothetical protein | -1.57 | 0.0003 |
|  | PA3416 | Pyruvate dehydrogenase E1 component beta subunit | -1.57 | 0.004 |
|  | *fdhE* | Formate dehydrogenase subunit epsilon | -1.57 | 0.02 |
|  | *NalD* (PA3574) | TetR/AcrR family transcriptional regulator, repressor of the mexAB-oprM multidrug resistance | -1.57 | 0.0001 |
|  | PA5325 | Hypothetical protein | -1.57 | 0.003 |
|  | PA1933 | Xanthine dehydrogenase YagR molybdenum-binding subunit | -1.57 | 0.0008 |
|  | PA4156 | Iron complex outermembrane recepter protein (TonB-dependent receptor) | -1.57 | 9.38E-07 |
|  | PA2701 | Major facilitator superfamily transporter | -1.57 | 0.04 |
|  | *xenB* | Xenobiotic reductase | -1.57 | 2.74E-07 |
|  | *iscU* | Nitrogen fixation protein NifU and related proteins | -1.56 | 0.0001 |
|  | PA3069 | Hypothetical protein | -1.56 | 4.42E-11 |
|  | PA1598 | 3-methyl-2-oxobutanoate hydroxymethyltransferase | -1.56 | 0.02 |
|  | *flgE* | Flagellar hook protein FlgE | -1.55 | 0.002 |
|  | *pscT* | Type III secretion protein T | -1.55 | 0.001 |
|  | PA4994 | Acyl-CoA dehydrogenase | -1.54 | 0.02 |
|  | PA4518 | Hypothetical protein | -1.54 | 0.01 |
|  | *yfiR* | yfiR; hypothetical protein (Repressor protein) | -1.54 | 0.004 |
|  | PA2875 | MoxR-like ATPase | -1.53 | 0.01 |
|  | *oprG* | Outer membrane protein OprG | -1.53 | 7.81E-09 |
|  | PA1506 | Hypothetical protein | -1.53 | 0.03 |
|  | PA1416 | Hypothetical protein | -1.53 | 0.0001 |
|  | *yrfI* | molecular chaperone Hsp33 (heat shock protein 33) | -1.53 | 0.001 |
|  | PA2485 | Hypothetical protein | -1.53 | 0.03 |
|  | *fliE* | Flagellar hook-basal body complex protein FliE | -1.53 | 1.54E-06 |
|  | PA1623 | GSH-dependent disulfide-bond oxidoreductase | -1.53 | 0.006 |
|  | *alg*8 | Mannuronan synthase (Glycosyltransferase) | -1.52 | 0.008 |
|  | *pilU* | Twitching motility protein | -1.52 | 4.41E-06 |
|  | PA5246 | Hypothetical protein | -1.52 | 0.02 |
|  | PA4478 | Septum formation protein | -1.52 | 0.0007 |
|  | PA4716 | Hypothetical protein | -1.52 | 1.04E-06 |
|  | PA2151 | Starch synthase (maltosyl-transferring) | -1.52 | 0.02 |
|  | PA0727 | Hypothetical protein | -1.52 | 0.009 |
|  | PA0243 | Transcriptional regulator | -1.52 | 0.02 |
|  | PA0714 | Hypothetical protein | -1.52 | 0.05 |
|  | PA0729 | Hypothetical protein | -1.52 | 0.02 |
|  | PA4734 | Hypothetical protein | -1.52 | 0.003 |
|  | *mexG* | Transmembrane protein | -1.52 | 0.02 |
|  | PA2079 | Amino acid permease | -1.52 | 0.01 |
|  | PA3180 | 8-oxo-dGTP diphosphatase | -1.51 | 0.001 |
|  | PA2877 | Transcriptional regulator | -1.51 | 0.01 |
|  | PA5126 | Hypothetical protein | -1.51 | 0.0007 |
|  | PA4219 | Hypothetical protein | -1.51 | 0 |
|  | *pauA* | Pimeloyl-CoA synthetase | -1.51 | 0.02 |
|  | PA2265 | Gluconate 2-dehydrogenase alpha chain | -1.51 | 0.02 |
|  | PA5446 | Hypothetical protein | -1.51 | 1.8E-09 |
|  | *ptsN* | PTS system, nitrogen regulatory IIA component | -1.51 | 0.005 |
|  | PA0204 | Spermidine/putrescine transport system permease protein  (ABC transporter permease) | -1.51 | 0.03 |
|  | PA4878 | Transcriptional regulator | -1.50 | 0.001 |
|  | PA1382 | Type II secretion system protein | -1.50 | 0.0008 |
|  | *rsaL* | Regulatory protein RsaL | -1.50 | 7.57E-05 |
|  | *glpK* | Glycerol kinase | -1.50 | 0.0003 |
|  | PA4506 | Dipeptide transport system ATP-binding protein | -1.50 | 0.0001 |
|  | PA2481 | Thiosulfate dehydrogenase | -1.50 | 7.04E-07 |
|  | PA4791 | Hypothetical protein | -1.50 | 0.005 |
|  | PA4460 | Lipopolysaccharide export system protein LptA | -1.50 | 0.001 |
|  | PA0554 | Hypothetical protein | -1.50 | 0.001 |
|  | PA1439 | Hypothetical protein | -1.50 | 0.03 |
|  | PA2723 | Hypothetical protein | -1.50 | 0.05 |

**Table S2: List of up-regulated genes**

| **Sr. No.** | **Feature ID/Gene** | **Coding for** | **Fold Change** | **P-value** |
| --- | --- | --- | --- | --- |
|  | *mexC* | Membrane fusion protein, multidrug efflux system | 16.82 | 0 |
|  | PA2139 | Pseudogene | 15 | 0.01 |
|  | PA3441 | Molybdopterin-binding protein | 10 | 0.006 |
|  | PA5328 | Mono-heme cytochrome C | 8 | 0.01 |
|  | PA2819.1 | tRNA-Gly | 7.50 | 0.02 |
|  | PA2161 | Hypothetical protein | 7.22 | 4.16E-06 |
|  | *oprJ* | Outer membrane protein, multidrug efflux system | 6.91 | 0 |
|  | PA3383 | Phosphonate transport system substrate-binding protein | 6.33 | 0.0006 |
|  | PA0700 | Hypothetical protein | 5.80 | 0.003 |
|  | PA2565 | Hypothetical protein | 5.74 | 0 |
|  | *opdE* | Transcriptional regulator | 5.66 | 0.02 |
|  | PA0909 | Hypothetical protein | 5.40 | 0.005 |
|  | *napB* | Cytochrome c-type protein | 5.26 | 1.12E-13 |
|  | PA2090 | Hypothetical protein | 5.11 | 0.0004 |
|  | PA2285 | Hypothetical protein | 4.80 | 1.55E-05 |
|  | *hpcD* | 5-carboxymethyl-2-hydroxymuconate isomerase | 4.80 | 0.01 |
|  | PA3566 | Hypothetical protein | 4.75 | 0.001 |
|  | PA0695 | Hypothetical protein | 4.42 | 0.005 |
|  | PA1107 | Diguanylate cyclase | 4.21 | 0 |
|  | *mexD* | Multidrug efflux pump | 4.15 | 0 |
|  | PA2364 | Type VI secretion system protein | 4.03 | 1.29E-12 |
|  | PA4866 | Phosphinothricin acetyltransferase | 4 | 0.002 |
|  | PA3442 | Sulfonate transport system ATP-binding protein | 4 | 0.004 |
|  | *coaB* | Phage coat protein B | 4 | 0.01 |
|  | PA1231 | Hypothetical protein | 3.93 | 0.0002 |
|  | PA4172 | Exodeoxyribonuclease III | 3.93 | 5E-07 |
|  | PA1478 | Heme exporter protein D | 3.57 | 0.02 |
|  | PA0640 | Bacteriophage protein | 3.55 | 1.84E-07 |
|  | PA2499 | Deaminase | 3.53 | 0.002 |
|  | PA1352 | Hypothetical protein | 3.51 | 1.34E-05 |
|  | *ospR* | Transcriptional regulator | 3.43 | 0 |
|  | *cdhA* | Carnitine 3-dehydrogenase | 3.42 | 0.002 |
|  | PA4908 | Ornithine cyclodeaminase | 3.31 | 1.18E-06 |
|  | PA3371 | Hypothetical protein | 3.22 | 0.02 |
|  | PA5391 | Hypothetical protein | 3.21 | 0.0008 |
|  | PA2307 | NitT/TauT family transport system permease protein | 3.18 | 8.69E-05 |
|  | PA0678 | General secretion pathway protein H (HxcU pseudopilin) | 3 | 0.03 |
|  | PA0684 | General secretion pathway protein M | 3 | 0.03 |
|  | PA2580 | Modulator of drug activity B | 3 | 0.03 |
|  | PA5135 | Hypothetical protein | 2.97 | 9.42E-06 |
|  | PA4290 | Methyl-accepting chemotaxis protein | 2.96 | 0 |
|  | PA3235 | Hypothetical protein | 2.96 | 1.62E-10 |
|  | PA2933 | large subunit ribosomal protein L6 (rplF; 50S ribosomal protein L6) | 2.96 | 0.0004 |
|  | PA3287 | Hypothetical protein | 2.92 | 5.2E-14 |
|  | PA0384 | Hypothetical protein | 2.92 | 0.01 |
|  | PA0848 | Peroxiredoxin (alkyl hydroperoxide reductase subunit C) | 2.90 | 2.08E-09 |
|  | PA1021 | enoyl-CoA hydratase | 2.88 | 0.0007 |
|  | PA2916 | Hypothetical protein | 2.86 | 0.009 |
|  | PA0633 | Hypothetical protein | 2.84 | 1.29E-10 |
|  | PA0638 | Bacteriophage protein | 2.84 | 8.11E-06 |
|  | PA1881 | Isoquinoline 1-oxidoreductase subunit alpha | 2.77 | 0.05 |
|  | *msuE* | FMN reductase | 2.75 | 0.01 |
|  | PA0623 | Bacteriophage protein | 2.74 | 9.84E-08 |
|  | *soxG* | Sarcosine oxidase | 2.73 | 0.002 |
|  | PA0814 | Hypothetical protein | 2.71 | 0.01 |
|  | PA2666 | 6-pyruvoyltetrahydropterin/6-carboxytetrahydropterin synthase | 2.70 | 0.009 |
|  | PA3431 | Hypothetical protein | 2.70 | 0.009 |
|  | PA5431 | GntR family transcriptional regulator | 2.67 | 1.41E-08 |
|  | PA0941 | Hypothetical protein | 2.66 | 0.01 |
|  | PA3757 | GntR family transcriptional regulator | 2.64 | 0.01 |
|  | PA0569 | Hypothetical protein | 2.63 | 0.04 |
|  | PA0915 | Hypothetical protein | 2.63 | 0.04 |
|  | PA2679 | Hypothetical protein | 2.61 | 0 |
|  | PA0622 | Bacteriophage protein | 2.59 | 3.12E-11 |
|  | PA1343 | Bacteriophage protein | 2.59 | 1.03E-08 |
|  | *mraY* | Phospho-N-acetylmuramoyl-pentapeptide-transferase | 2.59 | 1.92E-10 |
|  | PA0185 | Sulfonate transport system permease protein | 2.55 | 1.45E-06 |
|  | PA4596 | Transcriptional regulator | 2.50 | 3.21E-09 |
|  | PA1260 | Polar amino acid transport system substrate-binding protein | 2.50 | 0.01 |
|  | PA3606 | DTW domain-containing protein | 2.47 | 0.0001 |
|  | PA1977 | Hypothetical protein | 2.47 | 1.73E-05 |
|  | *vqsM* | HTH-type transcriptional regulator | 2.46 | 0.04 |
|  | *hutU* | Urocanate hydratase | 2.46 | 0 |
|  | *mdcC* | Malonate decarboxylase delta subunit | 2.45 | 0.009 |
|  | *hasD* | ATP-binding cassette, subfamily C, bacterial exporter for protease/lipase | 2.45 | 5.11E-08 |
|  | *betT1* | Choline/glycine/proline betaine transport protein | 2.44 | 0.0009 |
|  | *gloA2* | Lactoylglutathione lyase | 2.44 | 0.01 |
|  | *pslK* | Polysaccharide biosynthesis protein PslK | 2.44 | 2.46E-05 |
|  | *cysC* | Bifunctional enzyme CysN/CysC | 2.43 | 0.02 |
|  | PA2122 | Hypothetical protein | 2.43 | 0.0002 |
|  | *ahpC* | Peroxiredoxin (alkyl hydroperoxide reductase subunit C) | 2.43 | 0 |
|  | PA3412 | Hypothetical protein | 2.42 | 0.01 |
|  | PA3938 | Taurine transport system substrate-binding protein | 2.41 | 0.002 |
|  | PA1038 | Hypothetical protein | 2.40 | 0.005 |
|  | PA1958 | Nicotinamide mononucleotide transporter | 2.39 | 0.0004 |
|  | PA5377 | Glycine betaine/proline transport system permease protein | 2.39 | 0.0002 |
|  | PA0480 | 3-oxoadipate enol-lactonase | 2.38 | 0.05 |
|  | PA4093 | Hypothetical protein | 2.37 | 0.009 |
|  | PA2045 | Membrane protein insertion efficiency factor | 2.36 | 0.02 |
|  | PA1518 | 5-hydroxyisourate hydrolase | 2.36 | 0.01 |
|  | *ureD* | Urease accessory protein | 2.35 | 0.003 |
|  | PA3367 | Hypothetical protein | 2.35 | 0.03 |
|  | PA0118 | Hypothetical protein | 2.33 | 0.01 |
|  | PA5033 | Hypothetical protein | 2.32 | 0.001 |
|  | PA3453 | Hypothetical protein | 2.32 | 1.43E-07 |
|  | *mtlD* | Mannitol 2-dehydrogenase | 2.32 | 0.01 |
|  | PA1473 | Flagellar biosynthesis protein | 2.31 | 0.02 |
|  | PA1508 | Hypothetical protein | 2.31 | 0.02 |
|  | PA2352 | Glycerophosphoryl diester phosphodiesterase | 2.30 | 0.0008 |
|  | PA3534 | Oxidoreductase | 2.30 | 1.04E-06 |
|  | PA0962 | Starvation-inducible DNA-binding protein | 2.28 | 4.83E-09 |
|  | *hcnA* | Hydrogen cyanide synthase | 2.27 | 0.01 |
|  | *mobA* | Molybdenum cofactor guanylyltransferase | 2.27 | 0.01 |
|  | PA2111 | Hypothetical protein | 2.26 | 6.66E-16 |
|  | PA1922 | Outer membrane receptor for ferrienterochelin and colicins | 2.25 | 0.01 |
|  | PA4578 | Hypothetical protein | 2.25 | 6.67E-08 |
|  | PA4790 | S-adenosylmethionine-dependent methyltransferase | 2.25 | 0.006 |
|  | PA4280.5 | 16S ribosomal RNA | 2.25 | 0 |
|  | PA0647 | Hypothetical protein | 2.25 | 0.001 |
|  | *acsA* | Acetyl-CoA synthetase | 2.24 | 0 |
|  | PA4773 | S-adenosylmethionine decarboxylase | 2.23 | 0.04 |
|  | PA4508 | Lrp/AsnC family transcriptional regulator, leucine-responsive regulatory protein | 2.22 | 0.002 |
|  | PA0098 | 3-oxoacyl-[acyl-carrier-protein] synthase I | 2.21 | 0.003 |
|  | PA4651 | Fimbrial chaperone protein | 2.19 | 0.001 |
|  | PA2715 | Ferredoxin | 2.19 | 0.03 |
|  | PA2555 | Acetyl-CoA synthetase | 2.17 | 8.88E-16 |
|  | *xcpT* | Type II secretion system protein G | 2.17 | 0.002 |
|  | PA0621 | Hypothetical protein | 2.16 | 0.02 |
|  | PA0817 | Hypothetical protein | 2.16 | 0.01 |
|  | *masA* | Enolase-phosphatase E1 | 2.15 | 2.3E-11 |
|  | PA2375 | Hypothetical protein | 2.15 | 0.003 |
|  | PA2826 | Glutathione peroxidase | 2.14 | 1.89E-07 |
|  | PA5445 | Succinyl-CoA:acetate CoA-transferas | 2.13 | 4.02E-09 |
|  | PA0630 | Hypothetical protein | 2.11 | 0.009 |
|  | PA0557 | Hypothetical protein | 2.10 | 0.0009 |
|  | *panD* | Aspartate 1-decarboxylase | 2.10 | 0.006 |
|  | PA1424a | Hypothetical protein | 2.10 | 0.05 |
|  | PA2280 | Arsenical resistance protein ArsH | 2.10 | 0.05 |
|  | *cyoA* | Cytochrome o ubiquinol oxidase subunit II | 2.09 | 0.003 |
|  | *nuoK* | NADH-quinone oxidoreductase subunit K | 2.08 | 0.03 |
|  | PA0385 | Hypothetical protein | 2.08 | 0.02 |
|  | PA0306a | Transcriptional regulator | 2.07 | 3.45E-05 |
|  | PA0107 | Cytochrome c oxidase assembly protein subunit 11 | 2.07 | 0.02 |
|  | *oprH* | oprH; PhoP/Q and low Mg^2+^ inducible outer membrane protein H1 | 2.06 | 0 |
|  | *rpmD* | Large subunit ribosomal protein L30 | 2.05 | 4.8E-09 |
|  | PA3294 | Type VI secretion system secreted protein VgrG | 2.05 | 0.0002 |
|  | PA2293 | Hypothetical protein | 2.05 | 0.007 |
|  | PA3694 | Hypothetical protein | 2.05 | 0.007 |
|  | PA3289 | Hypothetical protein | 2.04 | 0.0009 |
|  | PA3568 | Propionyl-CoA synthetase | 2.03 | 2.01E-09 |
|  | PA5539 | GTP cyclohydrolase I | 2.03 | 0.01 |
|  | PA3420 | Transcriptional regulator | 2.03 | 2.15E-06 |
|  | PA0864 | Transcriptional regulator | 2.03 | 0.01 |
|  | *ampDh3* | N-acetylmuramoyl-L-alanine amidase | 2.02 | 0.008 |
|  | PA4612 | Hypothetical protein | 2.00 | 4.55E-07 |
|  | PA4824 | Hypothetical protein | 2 | 0.002 |
|  | PA3882 | Hypothetical protein | 2 | 0.006 |
|  | PA3332 | Hypothetical protein | 2 | 0.01 |
|  | *chiC* | Chitinase | 1.98 | 9.55E-05 |
|  | *bfrB* | Bacterioferritin | 1.98 | 9.48E-06 |
|  | PA1344 | Short-chain dehydrogenase | 1.97 | 0 |
|  | PA4832 | Short-chain dehydrogenase | 1.97 | 0.007 |
|  | *phzC1* | 3-deoxy-7-phosphoheptulonate synthase (phzC1; phenazine biosynthesis) | 1.97 | 0 |
|  | PA0618 | Bacteriophage protein | 1.97 | 1.85E-05 |
|  | PA4141 | Hypothetical protein | 1.96 | 0 |
|  | PA1665 | Type VI secretion system protein ImpI | 1.95 | 1.89E-05 |
|  | PA0398 | Hypothetical protein | 1.94 | 0.0008 |
|  | PA2525 | Hypothetical protein | 1.93 | 0.0004 |
|  | PA3205 | Hypothetical protein | 1.93 | 0.007 |
|  | PA0352 | Xanthine permease XanP | 1.93 | 0.0003 |
|  | *sucD* | Succinyl-CoA synthetase alpha subunit | 1.93 | 0 |
|  | PA1877 | Membrane fusion protein, adhesin transport system | 1.92 | 0.0005 |
|  | PA0532 | Hypothetical protein | 1.92 | 0.04 |
|  | PA2359 | Sigma-54 dependent transcriptional regulator | 1.92 | 0.0002 |
|  | PA3492 | Electron transport complex protein RnfD | 1.92 | 0.002 |
|  | PA2404 | Hypothetical protein | 1.91 | 0.02 |
|  | PA4380 | Two-component sensor | 1.91 | 0.001 |
|  | PA0187 | Hypothetical protein | 1.91 | 0.02 |
|  | PA1142 | Transcriptional regulator | 1.91 | 0.0007 |
|  | PA1351 | RNA polymerase sigma-70 factor, ECF subfamily | 1.90 | 0.03 |
|  | PA4088 | Glutamate-1-semialdehyde 2,1-aminomutase | 1.90 | 0.03 |
|  | *ahpF* | Alkyl hydroperoxide reductase subunit F | 1.89 | 0 |
|  | PA5357 | Chorismate--pyruvate lyase | 1.89 | 0.01 |
|  | PA4711 | Hypothetical protein | 1.88 | 0.002 |
|  | PA4202 | Nitronate monooxygenase | 1.88 | 0.0003 |
|  | *aruI* | 5-guanidino-2-oxopentanoate decarboxylase | 1.87 | 4.52E-05 |
|  | PA1265 | Hypothetical protein | 1.87 | 0.01 |
|  | PA3295 | Histidine triad (HIT) family protein | 1.86 | 0.008 |
|  | PA1851 | Hypothetical protein | 1.86 | 5.8E-06 |
|  | PA1566 | Glutamine synthetase | 1.86 | 0.009 |
|  | PA2205 | Hypothetical protein | 1.86 | 0.005 |
|  | *phoP* | Phosphopantothenoylcysteine decarboxylase / phosphopantothenate--cysteine ligase | 1.86 | 5.55E-15 |
|  | PA0631 | Hypothetical protein | 1.86 | 0.05 |
|  | PA5534 | Hypothetical protein | 1.85 | 0.004 |
|  | PA2441 | Hypothetical protein | 1.85 | 1.99E-08 |
|  | *katA* | Catalase | 1.85 | 0 |
|  | PA4097 | (R,R)-butanediol dehydrogenase / meso-butanediol dehydrogenase / diacetyl reductase | 1.84 | 0.04 |
|  | PA3301 | Hypothetical protein | 1.84 | 0.0007 |
|  | *liuA* | Isovaleryl-CoA dehydrogenase | 1.84 | 9.44E-11 |
|  | PA4298 | Hypothetical protein | 1.84 | 0.02 |
|  | PA3565 | Transcriptional regulator | 1.84 | 0.0003 |
|  | PA3674 | Putative lipoprotein | 1.83 | 0.05 |
|  | PA5144 | Hypothetical protein | 1.83 | 0.05 |
|  | PA0839 | TetR/AcrR family transcriptional regulator, transcriptional repressor for nem operon | 1.83 | 0.001 |
|  | *pasP* | Hypothetical protein (Small protease) | 1.82 | 3.37E-09 |
|  | PA0624 | Hypothetical protein | 1.82 | 0.001 |
|  | PA1916 | Amino acid permease | 1.82 | 0.02 |
|  | PA0356 | Hypothetical protein | 1.82 | 0.004 |
|  | *ccmE* | Cytochrome c-type biogenesis protein | 1.81 | 0.003 |
|  | *atuG* | Citronellol/citronellal dehydrogenase | 1.81 | 0.03 |
|  | *braE* | Branched-chain amino acid transport system permease protein | 1.81 | 0.008 |
|  | PA0274 | Hypothetical protein | 1.80 | 0.04 |
|  | PA2589 | Hypothetical protein | 1.80 | 0.04 |
|  | PA0556 | Hypothetical protein | 1.80 | 0.008 |
|  | PA0175 | Chemotaxis protein methyltransferase CheR | 1.80 | 0.03 |
|  | PA1894 | Hypothetical protein | 1.80 | 0.03 |
|  | PA1350 | Hypothetical protein | 1.80 | 0.04 |
|  | PA4849 | Hypothetical protein | 1.80 | 0.04 |
|  | PA4530 | Zinc-binding protein | 1.79 | 0.01 |
|  | PA1385 | Glycosyl transferase family protein | 1.79 | 0.01 |
|  | *flgF* | Flagellar basal-body rod protein | 1.79 | 0.0003 |
|  | PA0629 | Putative chitinase | 1.79 | 0.001 |
|  | PA2839 | 4,5-DOPA dioxygenase extradiol | 1.78 | 0.03 |
|  | PA4071 | Hypothetical protein | 1.78 | 0.03 |
|  | PA2949 | Lipase | 1.78 | 0.001 |
|  | PA3214 | Cholesterol transport system auxiliary component | 1.77 | 9.18E-07 |
|  | PA0643 | Hypothetical protein | 1.77 | 0.001 |
|  | PA3292 | Hypothetical protein | 1.77 | 0.008 |
|  | PA2990 | Glycerophosphoryl diester phosphodiesterase | 1.77 | 0.0006 |
|  | PA1280 | Alpha-ribazole phosphatase | 1.76 | 0.03 |
|  | PA2197 | Hypothetical protein | 1.76 | 0.03 |
|  | PA1908 | Major facilitator superfamily transporter | 1.76 | 0.007 |
|  | PA4912 | Branched-chain amino acid transport system permease protein | 1.76 | 0.01 |
|  | *cbpD* | Chitin-binding protein | 1.76 | 0.0003 |
|  | *mtlR* | Transcriptional regulator MtlR | 1.76 | 0.005 |
|  | PA1132 | Hypothetical protein | 1.75 | 0.005 |
|  | PA0745 | Enoyl-CoA hydratase | 1.75 | 0.01 |
|  | PA0307 | Hypothetical protein | 1.75 | 0.02 |
|  | PA1042 | Putative membrane protein | 1.75 | 0.04 |
|  | PA4108a | Hypothetical protein | 1.75 | 0.04 |
|  | PA0735 | Hypothetical protein | 1.75 | 0.05 |
|  | *phoA* | Alkaline phosphatase | 1.75 | 0.05 |
|  | *glmR* | Transcriptional regulator | 1.74 | 0.01 |
|  | *icp* | Inhibitor of cysteine peptidase | 1.74 | 0.01 |
|  | *lolA* | Outer membrane lipoprotein carrier protein | 1.74 | 0.0004 |
|  | PA0251 | Hypothetical protein | 1.73 | 0.01 |
|  | PA4453 | Phospholipid transport system substrate-binding protein | 1.73 | 8.21E-10 |
|  | PA5459 | Hypothetical protein | 1.73 | 0.004 |
|  | PA2857 | Putative ABC transport system ATP-binding protein | 1.72 | 0.002 |
|  | PA2112 | Hypothetical protein | 1.72 | 0.0003 |
|  | *rtcA* | RNA 3'-terminal phosphate cyclase (ATP) | 1.72 | 0.0003 |
|  | *sss* | Solute:Na+ symporter, SSS family (gpuP; 3-guanidinopropionate transporter) | 1.72 | 0.008 |
|  | PA0558 | Hypothetical protein | 1.71 | 0.002 |
|  | PA0756 | Two-component system, OmpR family, response regulator TctD | 1.71 | 0.004 |
|  | *waaA* | 3-deoxy-D-manno-octulosonic-acid (KDO) transferase | 1.71 | 3.1E-05 |
|  | PA1316 | Major facilitator superfamily transporter | 1.71 | 0.006 |
|  | PA2451 | Pseudogene | 1.71 | 0.0006 |
|  | PA3923 | Hypothetical protein | 1.71 | 7.34E-07 |
|  | PA0789 | amino acid transporter, AAT family (Amino acid permease) | 1.70 | 0.001 |
|  | PA0636 | Hypothetical protein | 1.70 | 5.94E-06 |
|  | *lptA* | Lipopolysaccharide export system protein LptA | 1.70 | 0.002 |
|  | PA3330 | Short-chain dehydrogenase | 1.70 | 3.75E-07 |
|  | PA1597 | Hypothetical protein | 1.69 | 1.08E-07 |
|  | *napF* | Ferredoxin-type protein NapF | 1.69 | 0.0005 |
|  | *phoR* | Two-component system, OmpR family, phosphate regulon sensor histidine kinase | 1.69 | 0.0001 |
|  | PA4317 | Hypothetical protein | 1.69 | 0.003 |
|  | *nfxB* | TetR/AcrR family transcriptional regulator, mexCD-oprJ operon repressor | 1.69 | 0.009 |
|  | PA1921 | Hypothetical protein | 1.69 | 0.01 |
|  | PA4899 | Aldehyde dehydrogenase (NAD+) | 1.69 | 0.001 |
|  | *chpB* | Chemosensory pili system protein | 1.68 | 0.0003 |
|  | PA1210 | Quercetin 2,3-dioxygenase | 1.68 | 0.01 |
|  | PA0879 | Acyl-CoA dehydrogenase | 1.68 | 0.02 |
|  | PA1223 | Transcriptional regulator | 1.68 | 0.005 |
|  | PA3855 | Hypothetical protein | 1.68 | 8.23E-05 |
|  | PA1033 | GSH-dependent disulfide-bond oxidoreductase | 1.68 | 0.03 |
|  | PA3018 | Hypothetical protein | 1.67 | 0.008 |
|  | PA4441 | Hypothetical protein | 1.67 | 0.008 |
|  | PA4149 | Hypothetical protein | 1.67 | 0.001 |
|  | *ccmG* | Cytochrome c biogenesis protein CcmG, thiol:disulfide interchange protein DsbE | 1.67 | 0.01 |
|  | *katN* | Mn-containing catalase | 1.67 | 0.05 |
|  | PA4801 | Hypothetical protein | 1.67 | 0.003 |
|  | PA1620 | Hypothetical protein | 1.67 | 0.03 |
|  | PA4579 | Hypothetical protein | 1.67 | 5.64E-05 |
|  | PA2475 | Cytochrome P450 | 1.66 | 0.003 |
|  | PA0608 | Phosphoglycolate phosphatase | 1.66 | 0.02 |
|  | PA4800 | Hypothetical protein | 1.65 | 0.05 |
|  | *xcpV* | General secretion pathway protein I | 1.65 | 0.05 |
|  | PA4690.2 | 23S ribosomal RNA | 1.65 | 0 |
|  | PA1824 | Hypothetical protein | 1.65 | 0.009 |
|  | PA1688 | Hypothetical protein | 1.65 | 0.002 |
|  | PA2281 | Transcriptional regulator | 1.65 | 0.01 |
|  | PA2554 | Short-chain dehydrogenase | 1.65 | 0.0004 |
|  | PA0660 | Nitronate monooxygenase | 1.65 | 0.0001 |
|  | PA2872 | Hypothetical protein | 1.65 | 2.87E-05 |
|  | PA0266 | 5-aminovalerate/4-aminobutyrate aminotransferase | 1.65 | 6.14E-06 |
|  | *pqqE* | Pyrroloquinoline quinone biosynthesis protein E | 1.65 | 0.001 |
|  | PA3730 | Hypothetical protein | 1.65 | 0.01 |
|  | PA4011 | Undecaprenyl-diphosphatase | 1.64 | 1.04E-06 |
|  | *psd* | Phosphatidylserine decarboxylase | 1.64 | 0.003 |
|  | PA4014 | Hypothetical protein | 1.64 | 0.05 |
|  | PA5522 | Glutamine synthetase | 1.64 | 0.0003 |
|  | PA1345 | Hypothetical protein | 1.64 | 0.004 |
|  | PA1662 | Type VI secretion system protein VasG | 1.64 | 2.08E-10 |
|  | *rbfA* | Ribosome-binding factor A \| | 1.63 | 0.03 |
|  | *stp1* | Serine/threonine protein phosphatase | 1.63 | 0.0009 |
|  | *prfA* | Peptide chain release factor 1 | 1.63 | 0.0001 |
|  | *hutH* | Histidine ammonia-lyase | 1.62 | 4.05E-09 |
|  | *mdcA* | Malonate decarboxylase alpha subunit | 1.62 | 1.57E-07 |
|  | PA3271 | Two-component sensor | 1.62 | 2.83E-08 |
|  | PA1129 | Glutathione S-transferase fosA | 1.62 | 0.05 |
|  | PA1232 | Hypothetical protein | 1.62 | 5.08E-05 |
|  | *hasR* | Heme acquisition protein HasR | 1.62 | 1.15E-05 |
|  | PA4319 | Hypothetical protein | 1.62 | 0.03 |
|  | PA5542 | Hypothetical protein | 1.62 | 0.007 |
|  | PA1266 | Oxidoreductase | 1.61 | 0.007 |
|  | PA2609 | Hypothetical protein | 1.61 | 0.007 |
|  | PA1643a | Aldehyde-activating protein | 1.61 | 0.01 |
|  | *fdx1* | 4Fe-4S ferredoxin | 1.61 | 0.04 |
|  | PA3464 | Hypothetical protein | 1.61 | 0.0005 |
|  | PA3690 | Cd2+/Zn2+-exporting ATPase | 1.61 | 1.29E-09 |
|  | *spuF* | Putrescine transport system ATP-binding protein | 1.60 | 0.0008 |
|  | PA3293 | Hypothetical protein | 1.60 | 0.04 |
|  | PA4010 | DNA-3-methyladenine glycosylase | 1.60 | 2.65E-05 |
|  | PA5075 | Polar amino acid transport system permease protein | 1.59 | 0.003 |
|  | PA0089 | Type VI secretion system protein ImpH | 1.59 | 0.03 |
|  | *aprA* | Serralysin | 1.59 | 0.001 |
|  | PA1357 | Hypothetical protein | 1.59 | 0.008 |
|  | PA3944 | Hypothetical protein | 1.59 | 0.02 |
|  | *pilS* | Two-component system, NtrC family, sensor histidine kinase | 1.59 | 0.01 |
|  | PA5362 | Hypothetical protein | 1.59 | 0.0004 |
|  | *ada* | Adenosine deaminase | 1.58 | 0.001 |
|  | PA0369 | Pseudogene | 1.58 | 0.01 |
|  | *retS* | Two-component system, sensor histidine kinase | 1.58 | 0.0002 |
|  | PA3234 | Cation/acetate symporter | 1.58 | 5.55E-05 |
|  | *accC* | Acetyl-CoA carboxylase, biotin carboxylase subunit | 1.58 | 1.51E-05 |
|  | PA2230 | Hypothetical protein | 1.58 | 0.01 |
|  | PA4591 | Hypothetical protein | 1.58 | 0.02 |
|  | *rrmA* | 23S rRNA (guanine745-N1)-methyltransferase | 1.58 | 0.02 |
|  | PA5064 | Ubiquinone biosynthesis protein UbiJ | 1.58 | 0.008 |
|  | PA5177 | 5'-nucleotidase | 1.58 | 0.008 |
|  | PA1766 | Hypothetical protein | 1.58 | 2.83E-06 |
|  | PA3080 | Hypothetical protein | 1.57 | 0.005 |
|  | PA1680 | Hypothetical protein | 1.57 | 0.01 |
|  | *glmS* | Glutamine--fructose-6-phosphate aminotransferase | 1.57 | 0.0003 |
|  | PA3213 | Phospholipid/cholesterol/gamma-HCH transport system substrate-binding protein | 1.57 | 0.008 |
|  | PA2870 | Hypothetical protein | 1.57 | 4.16E-06 |
|  | PA2539 | Hypothetical protein | 1.57 | 0.02 |
|  | *lasI* | Acyl homoserine lactone synthase | 1.57 | 0.0002 |
|  | *cobI* | Precorrin-2/cobalt-factor-2 C20-methyltransferase | 1.57 | 0.001 |
|  | PA3693 | Hypothetical protein | 1.57 | 0.008 |
|  | *phoQ* | Two-component system, OmpR family, sensor histidine kinase | 1.57 | 1.81E-12 |
|  | PA3784 | Hypothetical protein | 1.57 | 0.0006 |
|  | PA0641 | Bacteriophage protein | 1.57 | 9.27E-06 |
|  | PA2330 | Hypothetical protein | 1.56 | 1.79E-08 |
|  | PA0419 | 16S rRNA (uracil1498-N3)-methyltransferase | 1.56 | 0.01 |
|  | PA1852 | Hypothetical protein | 1.56 | 8E-05 |
|  | PA2540 | Hypothetical protein | 1.56 | 0.002 |
|  | PA4955 | Hypothetical protein | 1.55 | 0.001 |
|  | *tonB2* | Periplasmic protein TonB | 1.55 | 0.02 |
|  | PA0225 | Transcriptional regulator | 1.55 | 0.01 |
|  | PA1305 | Hypothetical protein | 1.55 | 0.01 |
|  | PA0389 | Hypothetical protein | 1.55 | 0.0001 |
|  | PA2548 | Phosphate transport system substrate-binding protein | 1.55 | 0.03 |
|  | *eddA* | Alkaline phosphatase D | 1.55 | 0.007 |
|  | *pscJ* | Type III secretion protein J | 1.55 | 0.03 |
|  | *cyoB* | Cytochrome o ubiquinol oxidase subunit III | 1.55 | 0.001 |
|  | PA0635 | Hypothetical protein | 1.54 | 0.01 |
|  | PA5317 | Peptide/nickel transport system substrate-binding protein | 1.54 | 0.01 |
|  | PA4120 | AraC family transcriptional regulator, 4-hydroxyphenylacetate 3-monooxygenase operon regulator | 1.54 | 0.02 |
|  | PA2289 | Iron complex outermembrane recepter protein | 1.54 | 0.03 |
|  | PA3211 | Phospholipid/cholesterol/gamma-HCH transport system permease protein | 1.54 | 0.0003 |
|  | PA3427 | Short-chain dehydrogenase | 1.54 | 0.003 |
|  | PA4927 | Hypothetical protein | 1.54 | 2.99E-06 |
|  | PA3087 | Hypothetical protein | 1.54 | 0.001 |
|  | *braF* | Branched-chain amino acid transport system ATP-binding protein \| (RefSeq) braF; ABC transport | 1.53 | 0.02 |
|  | PA3123 | Hypothetical protein | 1.53 | 0.006 |
|  | *mifS* | Two-component system, NtrC family, C4-dicarboxylate transport sensor histidine kinase DctB | 1.53 | 0.013 |
|  | PA0626 | Hypothetical protein | 1.53 | 0.003 |
|  | *argA* | Amino-acid N-acetyltransferase | 1.53 | 2.7E-07 |
|  | *tesB* | Acyl-CoA thioesterase II | 1.53 | 0.03 |
|  | *pyrF* | Orotidine-5'-phosphate decarboxylase | 1.53 | 0.004 |
|  | PA1649 | Short-chain dehydrogenase | 1.52 | 0.04 |
|  | *glyA3* | Glycine hydroxymethyltransferase | 1.52 | 0.0001 |
|  | PA2116 | Hypothetical protein | 1.52 | 5.22E-05 |
|  | PA4143 | ATP-binding cassette, subfamily B, bacterial RaxB | 1.52 | 0.01 |
|  | PA0392 | YggT family protein | 1.52 | 0.02 |
|  | PA1668 | Type VI secretion system protein ImpK | 1.52 | 0.01 |
|  | *toxA* | Exotoxin A | 1.52 | 0.03 |
|  | PA0794 | 2-methylcitrate dehydratase (2-methyl-trans-aconitate forming) | 1.52 | 1.23E-06 |
|  | PA1051 | Gluconate:H+ symporter, GntP family | 1.52 | 0.02 |
|  | PA3073 | Ca-activated chloride channel homolog | 1.52 | 0.02 |
|  | *gltX* | Glutamyl-tRNA synthetase | 1.52 | 4.01E-06 |
|  | PA1194 | Arginine:ornithine antiporter / lysine permease | 1.52 | 0.04 |
|  | PA1960 | Hypothetical protein | 1.51 | 0.01 |
|  | PA0022 | L-threonylcarbamoyladenylate synthase | 1.51 | 0.0002 |
|  | *lpdG* | Dihydrolipoamide dehydrogenase | 1.51 | 4.83E-08 |
|  | PA4818 | Hypothetical protein | 1.51 | 0.03 |
|  | *leuB* | 3-isopropylmalate dehydrogenase | 1.51 | 0.007 |
|  | PA1396 | Two-component sensor | 1.50 | 0.01 |
|  | *ilvH* | Acetolactate synthase I/III small subunit | 1.50 | 0.02 |
|  | PA3602 | Hypothetical protein | 1.50 | 0.02 |
|  | PA2423 | Hypothetical protein | 1.50 | 0.01 |
|  | PA1441 | Flagellar hook-length control protein FliK | 1.50 | 0.0001 |
|  | *hcnC* | Hydrogen cyanide synthase | 1.50 | 1.21E-05 |
|  | *fliN* | Flagellar motor switch protein | 1.50 | 4.38E-05 |
|  | PA5390 | Acetylornithine deacetylase | 1.50 | 0.003 |
|  | PA0810 | 2-haloacid dehalogenase | 1.50 | 0.005 |
|  | PA1811 | Microcin C transport system substrate-binding protein | 1.50 | 0.006 |

**Table S3: Genes expressed exclusively in PF-exposed *P. aeruginosa* culture**

| **Up-regulated genes** | | | |
| --- | --- | --- | --- |
| **Sr. No.** | **Feature Id/ Gene** | **Function** | ***p-*value** |
| 1. | PA0722 | Hypothetical protein | 0.001 |
| 2. | PA2785 | Putative transcriptional regulator | 0.003 |
| 3. | PA3512 | NitT/TauT family transport system permease protein (ABC transporter permease) | 0.03 |
| 4. | *phzB2* | Phenazine biosynthesis protein | 0.003 |
| **Down-regulated genes** | | | |
| **Sr. No.** | **Feature Id/ Gene** | **Function** | ***p-*value** |
| 1. | PA2149 | Hypothetical protein | 0.01 |
| 2. | PA3824.1 | tRNA Leu | 0.02 |
| 3. | PA2174 | Hypothetical protein | 0.002 |
| 4. | PA2603.1 | tRNA Ser | 0.0009 |
| 5. | PA3133.2 | tRNA Ala | 0.01 |
| 6. | PA3262.2 | tRNA Val | 0.0009 |
| 7. | PA4277.1 | tRNA Thr | 0.01 |
| 8. | PA4746.1 | tRNA Met | 0.003 |

**Table S4: Category-wise list of genes expressed differentially in PF-treated *P. aeruginosa***

| **Genes related to** | **Up-regulated genes** | **Down-regulated genes** |
| --- | --- | --- |
| Chemotaxis / flagellar/ Twiching motility/ hook protein/ Biofilm | PA4290, PA0175, PA1473, *flgF, fliN*, PA2285, PA1441, PA3383, PA1107, PA1021 | *cheY, cheW*, *algE, flaG, flgE, fliE, pilU. YqgC,* PA5181.1, *narH, gcvA, pilC, yfiR,* PA2877, *cupB3* |
| Nitrosative stress response/ Nitrogen metabolism | *napB* | PA0521, *norB*, *norC*, PA0525, *nosY, nirQ, nirS, nirD, narH, ccoQ, dnr*, PA1334, *xenB, iscU, ptsN* |
| Oxidative stress response | PA4172, PA2580, *ospR* PA0848, *ahpC*, PA3534, PA2826, *nuoK*, PA1266, *katA, katN* | *nuoI*, PA0942, PA3450, PA1192, *yrfI* |
| Iron/ sulfur/ copper homeostasis | PA0848, PA1881, PA0185  *hasD*, PA1922, PA2715, PA1351, PA2289, PA5328, *hasR*  PA3442, *msuE, gloA2*, PA3938, PA1478 | PA4357, PA0665, *exbD1,* *bphO*, *fepB,* PA0192, PA1146, PA0242, *oprG*, PA4156, *nosL, nosY* |
| Molybdenum homeostasis | PA3441, *mobA* | *modA*, *,moeA1, moaC, moaE,* PA1933 |
| Dehydrogenases | *cdhA ,mtlD,* PA1344, PA4832, *liuA*, PA4097, *atuG*, PA3330, PA4899, PA0879, PA2554, PA3427, PA1649, *leuB* | *lldD*, PA1020, PA4100, PA4995, PA1446, *fdhE,* PA4994, PA2481 |
| Efflux /Transport | *mexC, oprJ, mexD*, PA5328, PA3383, PA3442, PA1478, PA2307, PA2933, *atsB*, PA1260, *hasD*, *betT1*, PA3938, PA1958, PA5377, PA1518, PA1922, PA1877, PA1351, *braE,* PA3214, PA4912, PA4453, PA2857, *sss,* PA0789, *spuF*, PA5075, PA3213, PA2548, PA5317, PA3211, *braF, miffs,* PA1811 | *cueR*, PA0218, PA0828,  PA4779, PA2206, PA5179,  PA1131, PA3522, PA5518, PA3678, PA5428, PA3521, PA0325, *metR*, PA3574, PA2701, PA0243, *mexG*, PA4878, *oprG* |
| Acyl- carrier related protein/ fatty acid metabolism | PA0098 | PA1827 (*fabG),* PA0182, *fabI*, PA1470, *lipA*, PA2801, PA5519, *rsaL,* PA3859 |
| Transcriptional regulators | *opdE*, *ospR*, PA5431, PA3757, PA4596, *vqsM*, PA4508, PA0306a, PA3420, PA0864, PA4612, PA2359, PA1142, PA3565, PA0839, *mtlR*, *glmR*, *nfxB*, PA1223, PA2281, PA0225, PA4120 | *ArsR*, PA1312, *gntR*, PA0942, PA0218, PA0828 (*LysR*)*,* PA1015 (*glyR*)*, ohrR*, PA2206, PA5179, PA5324 (*sphR)*, PA1184, PA3965, PA2588, PA2758, PA2047, PA0367, PA1141, PA4185, *fis, glpR*, PA2270, PA2930, PA3678, PA5428, *gpuR*, PA0120, PA1264, *metR,* PA0243 PA2877*,* PA1484, PA4878 (*brlR*) |
| Virulence | - | PA5071, PA2146, *algE*, PA4171, *glpD*, PA3913, *narL*, PA0277, PA0688 |
| Secretion | PA2364, PA0640, PA0678, PA0684, PA0638, PA0623, PA0622, PA1343, PA1473, *xcpT*, PA0168, PA3294, PA1665, PA0089, PA0641, *xcpV*, PA1662, *pscJ, ImpK* | PA1702, *pscL,* PA0682, PA0087, *cupB3,* PA2369, PA2373, PA3404, PA3402, *pscT,* PA1382 |
| Basic cellular process and metabolism | PA2666, PA3492, *gloA2, hpcD, cdhA, soxG, hutU, mdcC,* PA3938, PA1518, PA2352, *acsA,* PA4773, PA2555, *panD, aruI, phoP, mdcA, accC, pyrF, cdhA*, *ureD*, *acsA* | PA2162, PA2691, *ppgL, gltR,* PA2265, *pcaC,* PA3172, PA0119, *cynS, dctA,* PA0216, PA2079, PA2265, *glpK,* PA3377, PA1013.1, PA5155_,_ *braZ*, *aruH*, PA4913, PA0530, PA2041, *aphA, aph,* PA0120, PA2079, PA2607, PA2570.1, PA3580, *aat,* PA1192, PA1790, PA2606  *nuoI,* PA2630, *pcaH,, nadE,* PA3172, PA1507, PA2317, PA1334, PA3416, PA2151, *pauA,* PA2265, *lgt* |
| Quorum-sensing | *lasI, phzC1* | *kynB,* PA5071 |

**Table S5: A comparison of efficacy of *Panchvalkal* when it is used on *P. aeruginosa* before infecting *C. elegnas*, versus, when it is used on *C. elegans* already infected by *P. aeruginosa* having no previous exposure to the *Panchvalkal* extract**

| **Mode of PF application** | **% Survival of worm population at the end of 5-day experiment**  **(Mean ± SD)** | **Net survival benefit at the end of 5-day experiment** (after nullifying natural death of few worms in ‘control’ wells)  **(Mean ± SD)** |
| --- | --- | --- |
| Pre-treatment of bacteria before allowing them to infect *C. elegans* | 90*** ± 3.55 | 68 ± 3.19 |
| PF used as a therapy for already infected *C. elegans* | 85*** ± 3.61 | 62.5 ± 0.92 |

PF: *Panchvalkal* formulation; Concentration of PF used in these experiments was 750 µg/mL.

Differences in the survival values for both modes of PF-application were statistically not significant (*p* value 0.16).
